# Single cell transcriptome analysis of the THY-Tau22 mouse model of Alzheimer’s disease reveals sex-dependent dysregulations

**DOI:** 10.1038/s41420-024-01885-9

**Published:** 2024-03-07

**Authors:** Muhammad Ali, Pierre Garcia, Laetitia P. Lunkes, Alessia Sciortino, Melanie Thomas, Tony Heurtaux, Kamil Grzyb, Rashi Halder, Djalil Coowar, Alex Skupin, Luc Buée, David Blum, Manuel Buttini, Enrico Glaab

**Affiliations:** 1 Luxembourg Centre for Systems Biomedicine (LCSB), University of Luxembourg, 7 avenue des Hauts Fourneaux, L-4362 Esch-sur-Alzette, Luxembourg; 2 Department of Life Sciences and Medicine (DLSM), University of Luxembourg, 8 avenue du Swing, L-4367 Belvaux, Luxembourg; 3 Luxembourg Center of Neuropathology, L-3555 Dudelange, Luxembourg; 4 University of Lille, Inserm, CHU Lille, UMR-S1172 Lille Neuroscience & Cognition (LilNCog), Lille, France; 5 Alzheimer and Tauopathies, LabEx DISTALZ, Lille, France

**Keywords:** Alzheimer's disease, Transcriptomics

## Abstract

Alzheimer’s disease (AD) progression and pathology show pronounced sex differences, but the factors driving these remain poorly understood. To gain insights into early AD-associated molecular changes and their sex dependency for tau pathology in the cortex, we performed single-cell RNA-seq in the THY-Tau22 AD mouse model. By examining cell type-specific and cell type-agnostic AD-related gene activity changes and their sex-dimorphism for individual genes, pathways and cellular sub-networks, we identified both statistically significant alterations and interpreted the upstream mechanisms controlling them. Our results confirm several significant sex-dependent alterations in gene activity in the THY-Tau22 model mice compared to controls, with more pronounced alterations in females. Both changes shared across multiple cell types and cell type-specific changes were observed. The differential genes showed significant over-representation of known AD-relevant processes, such as pathways associated with neuronal differentiation, programmed cell death and inflammatory responses. Regulatory network analysis of these genes revealed upstream regulators that modulate many of the downstream targets with sex-dependent changes. Most key regulators have been previously implicated in AD, such as *Egr1*, *Klf4*, *Chchd2*, complement system genes, and myelin-associated glycoproteins. Comparing with similar data from the Tg2576 AD mouse model and human AD patients, we identified multiple genes with consistent, cell type-specific and sex-dependent alterations across all three datasets. These shared changes were particularly evident in the expression of myelin-associated genes such as *Mbp* and *Plp1* in oligodendrocytes. In summary, we observed significant cell type-specific transcriptomic changes in the THY-Tau22 mouse model, with a strong over-representation of known AD-associated genes and processes. These include both sex-neutral and sex-specific patterns, characterized by consistent shifts in upstream master regulators and downstream target genes. Collectively, these findings provide insights into mechanisms influencing sex-specific susceptibility to AD and reveal key regulatory proteins that could be targeted for developing treatments addressing sex-dependent AD pathology.

## Introduction

Pronounced sex differences are evident in the pathology and clinical presentation of Alzheimer’s disease (AD), but the factors driving these differences remain poorly understood. Approximately 65% of Alzheimer’s disease (AD) diagnoses occur in females [1], and while this can be partly explained by a longer average life expectancy, females also have a higher age-adjusted prevalence of AD and a faster cognitive decline in AD than males, even when Aβ burden is similar in both sexes [2–4]. In addition, females display a higher tau tangle density even when the age and years of education are taken into account [4, 5]. Furthermore, the strongest genetic risk factor, the apolipoprotein E (*APOE*) ε4 allele, is associated with a higher risk of AD in females than in males [6, 7]. Although changes related to hormone levels, in particular the decline in progesterone following menopause, could potentially contribute to differences in AD progression between the sexes [8–10], a comprehensive assessment of the molecular factors that influence and mediate sex-dimorphic patterns in AD is still lacking. A more complete understanding of the mechanisms involved could not only improve our knowledge of the pathogenesis and progression of AD, but also provide the basis for the development of more effective, personalized or sex-stratified therapeutic approaches.

Previous research has suggested that the diversity of cellular and molecular factors involved in AD pathogenesis [11, 12] and the dysfunction of brain immune cells play a key role in the sex-specific pathophysiology of AD [13]. However, the complexity of sex-specific mechanisms at the single-cell level often remains hidden in conventional RNA sequencing (RNA-seq) studies of bulk brain tissue. Recent advances in single-cell RNA-Seq technology provide a new way to study how Alzheimer’s pathology affects specific brain cell types at the molecular level. Traditional genomic studies have primarily focused on diverse cell groups, making it difficult to discern the unique characteristics of individual cells. However, the advent of high-throughput methods for single-cell and single-nucleus analysis has enabled researchers to delve into specific cell subgroups, providing new insights into how gene expression changes within disease models or human tissues. By examining sample heterogeneity and disease-specific transcriptomic signatures in relevant cell types, it has become possible to comprehensively map the transcriptional spectrum, delineate their unique signaling pathways, and pinpoint sex- or genotype-specific factors associated with AD development.

Recently, researchers have analyzed single-nucleus RNA-Seq (snRNA-Seq) datasets derived from multiple affected brain regions in AD, including data from the entorhinal [14] and prefrontal [15] cortices of human AD patients and cognitively normal controls who were matched for age and sex. Grubman et al. performed an analysis of snRNA-seq data sourced from the entorhinal cortex of 12 AD individuals and controls matched for age and sex. Although they included sex as a covariate in their differential gene expression analysis model, they did not perform a specific analysis to explore sex-related differences in AD [14]. Similarly, Mathys et al. used single-cell RNA-seq (scRNA-seq) datasets derived from the prefrontal cortex of individuals with varying degrees of Aβ burden. Their work offered first insights into the overarching patterns of sexually distinct transcriptional responses to AD pathology in different cell types in the brain [15]. A recent study [16] showed that microglia from human Amyloid Precursor Protein (APP)-transgenic mice with high levels of amyloid beta, but not from hTau transgenic mice (same model as used here), exhibited a strong transcriptomic response involving many AD risk genes, but that study used only male mice. Overall, transcriptional changes specific to different sexes and distinct brain cell types, as well as their coordinated alterations in cellular pathways and mechanistic interrelationships in molecular sub-networks have not yet been fully explored.

A general limitation in the study of AD pathophysiology using human biospecimens is that the access to human brain tissue samples is typically limited to *post-mortem* stages, and peripheral tissues available during the early pre-symptomatic stages of AD often do not provide a sufficiently detailed and accurate surrogate measure of the pathological changes occurring in the brain. To overcome these limitations, murine models of AD offer a valuable complementary resource that allows a comprehensive examination of pathological changes in the brain during the initial, pre-symptomatic phase of the disease. In addition, animal models enable a more time- and cost-efficient study of age-related changes during disease progression.

While previous scRNA-seq studies have examined transcriptomic changes in APP-transgenic mice, including our study of Abeta-related pathology in the Tg2576 mouse model [17], cell type-specific and network-level molecular alterations in tau-based models remain underexplored, particularly regarding sex differences occurring already at early pathological stages. To address this gap and gain insights into sex-dependent molecular factors influencing susceptibility and onset of tau pathology, we have performed scRNA-seq profiling of the THY-Tau22 transgenic mouse model of tau pathology [18] at an early stage of disease initiation. This transgenic line is characterized by the over-expression of human 1N4R tau levels due to two specific genetic mutations (G272V and P301S). The neuron-specific Thy1.2 promoter selectively drives the neuronal over-expression of the mutated tau protein, leading to the development of tau pathology in the hippocampus and to a lesser extent in the cortex of the mouse brain. Starting at 3 months of age and continuing until they reach 10–12 months, mice experience a gradual decline in cognitive function, marked by synaptic dysfunction and neuroinflammation [19–21]. For the present study, and because we were interested in early stages of the disease, we characterized sex-dependent transcriptomic changes in the THY-Tau22 AD mouse model by performing scRNA-seq experiments in both transgenic and wild-type control mice in the cortex at 7 months age (we note that tau pathology is significantly less pronounced in the cortex than in the hippocampus). To the best of our knowledge, this is the first study that provides a detailed examination of sex-specific transcriptomic changes in the THY-Tau22 mouse model. By focusing on an early stage of pathology, our analyses may also inform the discovery of biomarkers and therapeutic targets for early intervention.

The manuscript is structured into three main sections. In the “Materials and methods” section, we present a detailed description of our research methodology. In particular, we describe the composition and handling of the animals studied, the data processing, quality control, integration, clustering, cell type annotation, and the bioinformatic analysis approaches. Next, the results section presents the identified differentially expressed genes showing sex-neutral, sex-specific, or sex-dimorphic alteration patterns, the enriched pathways associated with known AD biology, the reconstructed gene regulatory networks, and comparisons with previous single-cell RNA-seq datasets for human AD and the Tg2576 model. Finally, the discussion section interprets these computational findings and relates them to current knowledge of sex differences and molecular mechanisms in AD.

In summary, our results emphasize that early stages of disease progression, before the development of extensive pathology, distinctive molecular changes manifest in specific brain cell types. These changes show distinct sex-specific patterns and are intricately interconnected at the level of cellular pathways and molecular sub-networks. The results also reveal common alterations between the THY-Tau22 model, the Tg2576 model, and human AD patient samples, in particular a consistent sex-dependent dysregulation of myelin-associated genes, suggesting impairment of myelin plasticity as an early event in AD-like pathologies. Moreover, our results uncover unique aspects of tau pathology not observed in APP-based models, such as the Tg2576 model, and reveal more female-specific than male-specific transcriptomic changes across all cell types. Collectively, our findings provide insights into potential mechanisms influencing differences in AD susceptibility and progression between males and females, and represent a first step towards identifying key regulators and processes that could serve as targets for the development of sex-specific diagnostics and more personalized, tailored therapies for AD.

## Results

### No sex difference in Thio-S positive inclusions load in 7-month-old THY-Tau22 mice

To assess tau inclusion load independently of the hyperphosphorylation state of transgenic tau, we assessed the inclusion load by using Thio-S, a fluorescent dye that binds to beta-pleated sheets of aggregated proteins. In 7-month-old THY-Tau22 mice, we observed no discernible sex differences in inclusion load (Suppl. Figure 1). The interplay between sex and tau load in humans is intricate. Evidence suggests that females have higher tau level in the CSF only in APOE4 carriers [22], whereas, in cognitively normal individuals, women start to have higher tau after 80 years of age [23]. Mice have only one ApoE isoform and the mice used in this cohort were in the initial phases of the disease, so the lack of observed differences in tau load between the sexes may be expected.

### Single-cell RNA-seq data clustering and cell type identification

**Fig. 1:**
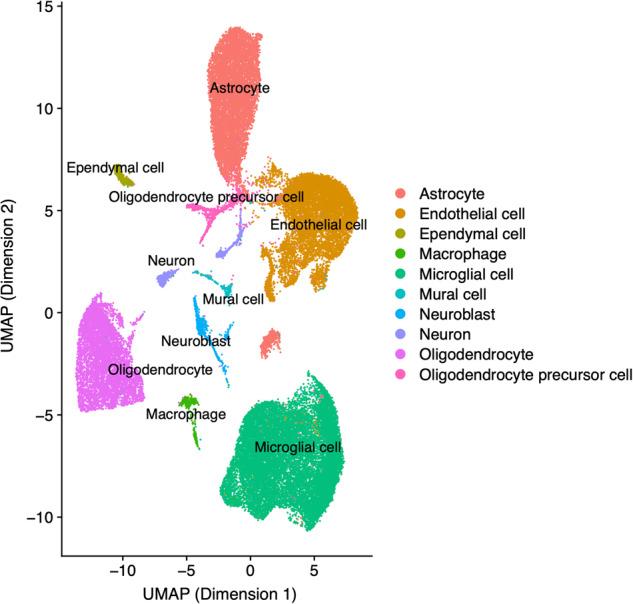
Cell type cluster visualization. The clusters of cells identified in the scRNA-seq data are visualized together with the cell type annotations using the Uniform Manifold Approximation and Projection (UMAP) method (covering the data from all mice across both sexes and conditions).

After pre-filtering and processing the raw read count data, we first identified clusters of cell types using the Shared Nearest Neighbor (SNN) modularity optimization-based clustering approach [24] (see “Materials and methods”). Selection of an appropriate number of clusters using the Silhouette Width score yielded the highest score (0.52) for 10 different clusters. A low-dimensional representation of these clusters was generated using the UMAP approach [25] and visualized together with their cell type annotations, determined as described below (see Fig. 1).

Cell type annotations obtained using the automated SCType approach [26] and marker genes from the CellMarker database [27] provided high-confidence cell type assignments for each major cell cluster. These clusters represent microglial cells (*n* = 16,769), oligodendrocytes (*n* = 6153), endothelial cells (*n* = 9204), astrocytes (*n* = 8605), oligodendrocyte precursor cells (OPCs; *n* = 961), macrophages (*n* = 505), ependymal cells (*n* = 474), mural cells (*n* = 347), neuroblasts (*n* = 748), and neurons (*n* = 816). Supplementary Table 1 provides more details on the corresponding cluster annotations and the marker genes used for each cluster. The differential expression patterns of these genes between the different cell type clusters are shown in the heat map in Suppl. Figure 2.

The observed modest representation of neuronal cells in the annotated data may be explained by the removal of myelin debris from single-cell suspensions as part of the experimental procedure, which results in neuronal loss [28]. However, the neuronal cell count remained adequate for robust statistical analyses, and the data captures other pivotal cell types, including microglia and astrocytes, which have been consistently associated with AD in numerous preceding studies. Specifically, microglial cells, which are the resident immune cells of the central nervous system, become activated in AD, contributing to chronic inflammation and neuronal damage [29], and are enriched in risk genes for late-onset AD [30]. Astrocytes show altered calcium signaling, glutamate transport, and antioxidant functions in AD models and patient samples, which disrupt neuronal support functions [31, 32]. Among additional cell types covered in our data, oligodendrocytes demonstrate compromised myelin integrity and repair capabilities in AD, resulting in white matter inflammation and loss of axonal support [33]. Endothelial cells of the cerebral microvasculature exhibit dysfunction of the blood-brain barrier in AD, enabling entry of toxic blood products into the brain [34]. Macrophages infiltrate the AD brain, where they secrete pro-inflammatory cytokines that propagate neuroinflammation [35]. In summary, the glial and vascular cell types present in our data are known to demonstrate functional impairments associated with pathological processes underlying AD.

### Analysis of sex-dependent differential gene expression in the THY-Tau22 model

To analyze differential gene expression between THY-Tau22 mice and wild-type controls, we took a dual approach: examining each cell type cluster individually (cell type-specific analysis) and assessing all clusters in aggregate (cell type-agnostic analysis). In addition, to discern the potential effect of sex on differential expression, we performed the differential gene expression analysis both for each sex independently and in a combined analysis with a dedicated interaction term for sex and genotype (see “Materials and methods”). In total, we identified 262 unique DEGs and 33 overlapping DEGs between these two approaches (Supplementary Tables 2 and 3). This allowed us to discern potential sex differences in the presence, magnitude and direction of significant transgene-associated changes. As described in the “Materials and methods” section, the DEGs identified for the first gene-level analysis approach were grouped into different categories, according to whether their patterns of change were sex-neutral (i.e., significant in both sexes, FDR < 0.05), sex-specific (significant in only one sex with FDR < 0.05, and not approaching significance in the other sex, nominal *p* > 0.5), or sex-dimorphic (significant in both sexes with FDR < 0.05, but with opposite direction of the log fold change (LFC) and a minimum LFC difference of 0.5). Summary statistics for the most significant sex-neutral, sex-specific, and sex-dimorphic DEGs are shown in Tables 1 to 3, with a full list of DEGs provided in Supplementary Table 2.

**Table 1:**
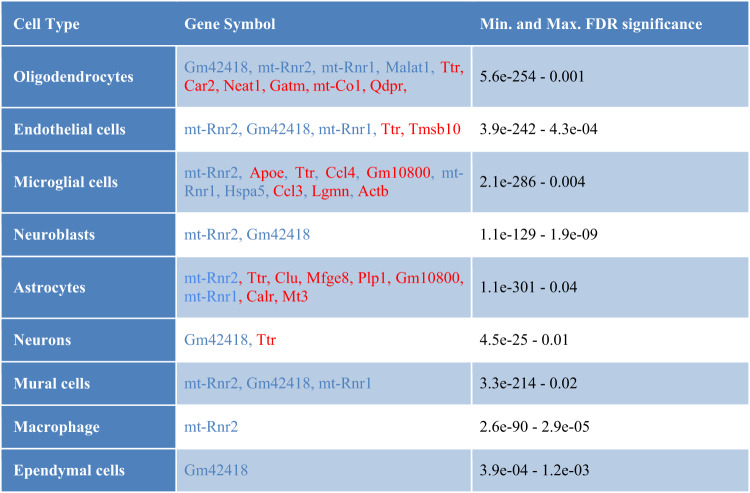
Sex-neutral DEGs.

Overview of the sex-neutral DEGs between transgenic and wild-type mice with highest significance (FDR < 0.05, up to 10 genes per cell type shown), i.e., representing overlapping DEGs between the sexes with the same direction of the transgene-associated alteration at the level of individual cell types (left column). Genes with increased expression (in THY-Tau22 mice compared to wild type) are depicted in red, while underexpressed genes are represented in blue.

**Table 2:**
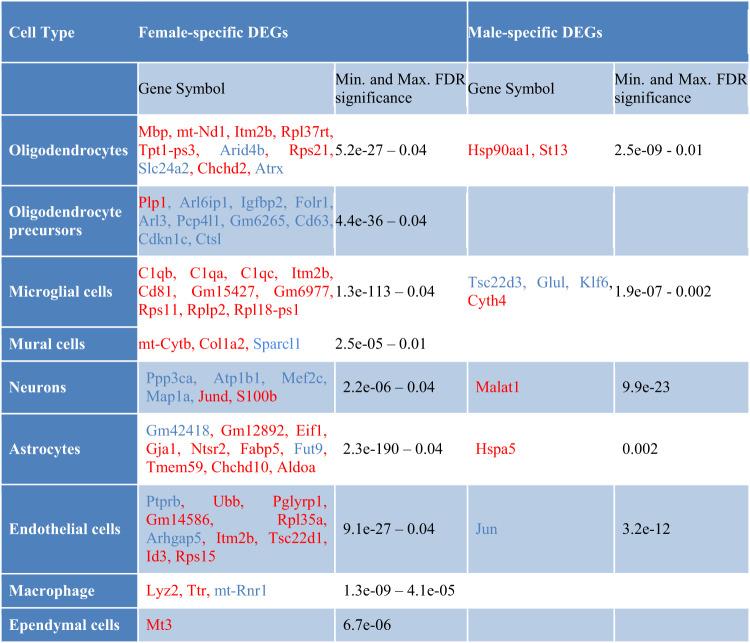
Sex-specific DEGs.

Overview of the sex-specific DEGs between transgenic and wild-type mice with highest significance (FDR < 0.05, up to 10 genes per cell type shown), i.e., representing the DEGs found to be significant in only one sex (FDR < 0.05), and which do not approach significance in the other sex (*p* > 0.5), studied at the level of individual cell types (left column). Genes with increased expression (in THY-Tau22 mice compared to wild type) are depicted in red, while underexpressed genes are represented in blue.

**Table 3:**
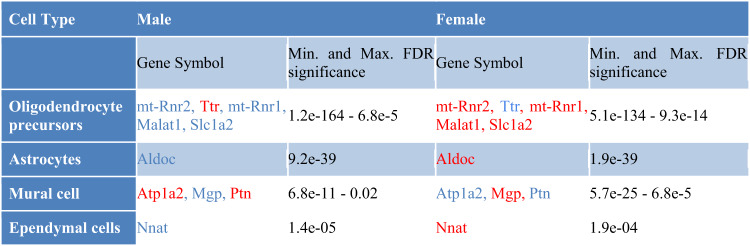
Sex-dimorphic DEGs.

Overview of the sex-dimorphic DEGs between transgenic and wild-type mice with highest significance (FDR < 0.05), i.e., representing the DEGs significantly differentially expressed in both sexes but with an opposite direction of the log fold change, studied at the level of individual cell types (left column). Genes with increased expression (in THY-Tau22 mice compared to wild type) are depicted in red, while underexpressed genes are represented in blue.

As a general observation, significant sex-dependent alterations were detected in most of the covered cell types, with large overlaps between the affected genes across these cell types. Specifically, we detected significant changes in multiple genes which had already displayed significant sex-neutral or sex-specific alterations in our previous study of the Tg2576 mouse model of Abeta pathology [17], including *mt-Rnr1, mt-Rnr2, Ttr, Slc1a2, Atp1a2, Mbp*, and *Malat1*. However, several new significant alterations specific to the THY-Tau22 model of tau pathology were also detected, such as changes in the genes *Aldoc*, *Mgp*, *mt-Nd1* (in the Tg2576 model, these genes reached neither adjusted nor nominal significance, with smallest nominal p-values of 0.11 for *Mgp* in endothelial cells and *p* = 0.09 for *mt-Nd1* in neurons; *Aldoc* was not detected in any of the cell type-specific differential analyses for this model).

Of particular interest is that, in contrast to findings reported in the Tg2576 AD mouse model [17], the THY-Tau22 mouse model showed more female-specific than male-specific transcriptomic alterations across all cell types. The most pronounced sex-specific changes were observed in microglial cells (68 female-specific DEGs, 4 male-specific DEGs) and endothelial cells (41 female-specific DEGs, 1 male-specific DEG; see also Supplementary Table 2). Overall, the analysis of sex-specific DEGs across all cell types revealed a total of 169 female-specific DEGs, 9 male-specific DEGs, 10 sex-dimorphic DEGs, and 47 sex-shared DEGs. While an in-depth analysis of each identified DEG exhibiting sex-dependent patterns is beyond the scope of this manuscript, Supplementary Table 4 provides an overview of the most significant genes with brief functional annotations in the context of AD.

Among the genes with the most pronounced alterations, the strongest sex-dimorphic changes were observed for *Aldoc*, which displayed a highly significant decreased expression in male astrocytes (FDR = 9.2E−39, logFC = −0.28), and a significantly increased expression in female astrocytes (FDR = 1.9E−39, logFC = 0.27). It encodes the protein aldolase C, which has been reported to undergo oxidation in the brains of individuals with mild cognitive impairment and AD [36]. This modification of aldolase C inhibits its activity and leads to an accumulation of fructose 1,6-bisphosphate, which promotes gluconeogenesis, inhibits glycolysis, and arrests the associated ATP production. Thus, the observed alterations in *Aldoc* gene expression may contribute to the dysregulation of glucose and energy metabolism associated with AD in a sex-specific manner, consistent with previous reports of sex-related differences in cerebral glucose metabolism [37] and particularly in the cortex in AD [38].

To identify additional sex-dependent changes in the magnitude of differences between transgenic and wild-type mice, we performed a further differential expression analysis of the THY-Tau22 scRNA-seq data for each cell type using an overdispersed Poisson model with an interaction term for sex and genotype (see “Materials and methods”). When comparing the analysis results using this approach with those from the sex-specific Seurat analysis, as expected, we identified common DEGs for most cell types, except for neurons, neuroblasts and ependymal cells (Suppl. Table 5). In addition, we found an overlap of sex-dimorphic genes for mural cells (*Atp1a2* and *Ptn*) and astrocytes (*Aldoc*). However, in line with the main purpose of this analysis to identify transgene-associated changes with sex-dependent differences in only the magnitude of the effects (and not the presence or direction of changes), we identified 295 new genes that displayed significant changes in the same direction across both sexes, but with a significant difference in the magnitude of the change. The top 5 significant DEGs from this analysis included *Gm42418* (FDR = 1.5E−151), *mt-Rnr2* (FDR = 1.9E−123), and *Malat1* (FDR = 5.5E−119), which are all altered significantly in the microglial cells, as well as *Ttr* (FDR = 3.5E−129) and *Aldoc* (FDR = 3.3E−86), identified in OPCs and astrocytes, respectively. The top 5 DEGs for each individual cell type are reported in Table 4, and the complete list of significant DEGs is provided in Supplementary Table 3.

**Table 4:**
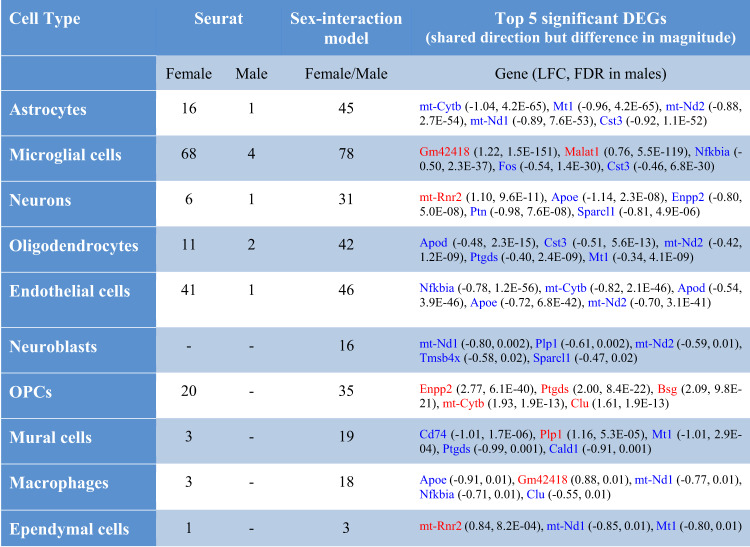
Overlap of DEGs between Seurat and edgeR approaches.

Over-expressed genes (in THY-Tau22 mice compared to wild type) are depicted in red, while under-expressed genes are represented by blue. Gene symbols and their log fold change (LFC) are reported for the top 5 significant DEGs with shared direction but difference in magnitude).

Overall, we identified both genes exhibiting significant sex-specific and sex-dimorphic patterns across multiple AD-relevant cell types, as well as further candidate sex-dependent genes that only displayed sex differences in the magnitude of changes between conditions. Many of these genes have known AD-associated functional annotations, which are further explored in the “Discussion” section.

### Cellular pathway analysis of sex-dependent differential gene expression

To investigate the global alterations in cellular processes in the cortex of THY-Tau22 mice, we conducted gene set enrichment analyses using the Gene Ontology (GO) database [39]. We initially performed a global combined analysis across all cell types to investigate the enrichment of genes with sex-dependent differential expression (cell type-agnostic analysis), followed by separate analyses for each individual cell type (cell type-specific analysis). Given the multitude of enriched pathways detected for individual cell types, discussing all results in detail is impractical. Therefore, we focus on elaborating the most robust findings from the global analysis. Pathway ranking tables for the two representative cell types with the largest numbers of measured cells, microglial and endothelial cells, are available in Supplementary Table 6. For determining sex-specificity, analogous to the gene-level analysis, we required a statistical significance of changes within one sex (FDR < 0.05) coupled with a clear absence of statistical significance in the other sex (*p* > 0.5). The specific input for the global cell type-agnostic pathway analysis included the identified 52 female-specific, 14 male-specific, and 44 sex-neutral DEGs (Suppl. Table 7). No sex-dimorphic DEGs were detected in this global analysis. Therefore, in the following we only discuss pathways enriched in sex-neutral DEGs (i.e., DEGs shared between the sexes) and sex-specific DEGs, and have highlighted the top five most enriched pathways from each category in Table 5 (the complete list of pathways is provided in the Suppl. Table 8).

**Table 5:** Biological pathway enrichment analysis.

Class	Category	ID	Description	Adj.P-value	Trend
Sex-neutral	BP	GO:0071674	Mononuclear cell migration	1.07E−04	Up
		GO:0060326	Cell chemotaxis	1.07E−04	Up
		GO:0098883	Synapse pruning	1.07E−04	Up
		GO:0042063	Gliogenesis	1.18E−04	Up
		GO:0030595	Leukocyte chemotaxis	1.18E−04	Up
	MF	GO:0004197	Cysteine-type endopeptidase activity	1.52E−03	Up
		GO:0004714	Transmembrane receptor protein tyrosine kinase activity	3.49E−03	Down
		GO:0008234	Cysteine-type peptidase activity	3.49E−03	Up
		GO:0042277	Peptide binding	3.71E−03	Up
		GO:0019199	Transmembrane receptor protein kinase activity	3.71E−03	Down
Female-specific	BP	GO:0046034	ATP metabolic process	1.98E−03	Up
		GO:0006091	Generation of precursor metabolites and energy	1.98E−03	Up
		GO:0045333	Cellular respiration	6.25E−03	Up
		GO:0010976	Positive regulation of neuron projection development	6.25E−03	Down
		GO:0006119	Oxidative phosphorylation	6.65E−03	Up
	MF	GO:0003735	Structural constituent of ribosome	5.27E−10	Up
		GO:0019843	rRNA binding	9.49E−06	Up
		GO:0015078	Proton transmembrane transporter activity	1.23E−03	Up
		GO:0046933	Proton-transporting ATP synthase activity, rotational mechanism	1.09E−02	Up
		GO:0015252	Proton channel activity	1.73E−02	Up
Male-specific	BP	GO:0002357	Defense response to tumor cell	6.13E−03	Equal
		GO:0001659	Temperature homeostasis	2.27E−02	Down
		GO:0048771	Tissue remodeling	2.27E−02	Up
		GO:1903320	Regulation of protein modification by small protein conjugation or removal	2.50E−02	Down
		GO:0002347	Response to tumor cell	2.50E−02	Equal
	MF	GO:1990841	Promoter-specific chromatin binding	2.78E−02	Down
		GO:0001221	Transcription coregulator binding	4.46E−02	Down
		GO:0140036	Ubiquitin-dependent protein binding	4.46E−02	Up
		GO:0019215	Intermediate filament binding	4.46E−02	Down
		GO:0004089	Carbonate dehydratase activity	4.46E−02	Up

Top 5 most significantly enriched GO terms (*BP* Biological Processes, *MF* Molecular Functions) with an overrepresentation of sex-neutral, female-specific, or male-specific DEGs. The column “Trend” reflects whether most of the DEGs in a particular pathway display increased (up) or decreased (down) expression levels, or whether equal numbers of increased and decreased DEGs occur in the pathway (equal).

#### Enrichment analysis of sex-neutral DEGs

**Fig. 2:**
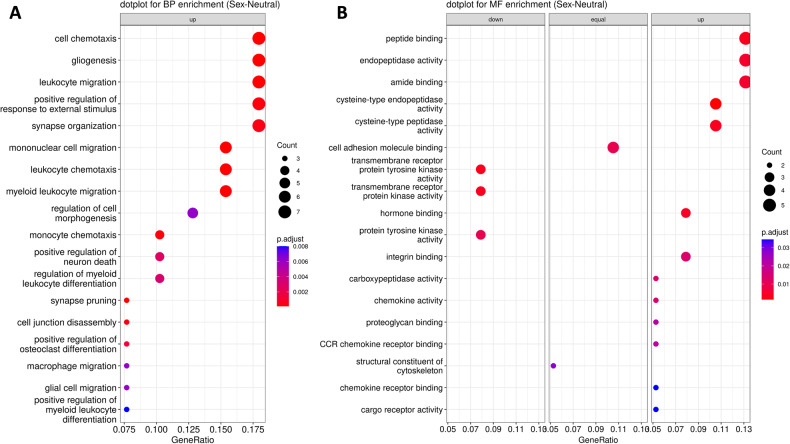
Dot plot visualization of sex-neutral changes in biological processes and molecular functions. Dot plot displaying the most significant Gene Ontology biological processes (BP) in (**A**), and molecular functions (MF) in (**B**), enriched in THY-Tau22-associated sex-neutral DEGs. The radius of the circles reflects the number of genes linked to specific GO terms, and the color gradient from red to blue reflects the adjusted *p*-value (p.adjust, see right side legend). The horizontal axis represents the gene ratio, indicating the extent of overlap between the input DEGs and the members of the pathway, relative to the overlap with all the members in the gene set collection. Wherever possible, each plot has been sub-categorized into three different pathway alteration trends, up, down, and equal, depending on whether the majority of DEGs displayed over-expression or under-expression, or whether an equal number of genes occurred in both categories.

Among the cellular processes significantly enriched in sex-neutral DEGs (FDR < 0.05), we noticed multiple cell migration-related processes, including the “leukocyte migration”, “mononuclear cell migration”, “macrophage migration”, and “glial cell migration” (see Fig. 2). This trend matched with notable alterations in chemotaxis-centric processes, including “cell chemotaxis”, “leukocyte chemotaxis”, and “monocyte chemotaxis”, as well as functions tied to chemokines, such as “chemokine activity”, “CCR chemokine receptor binding”, and “chemokine receptor binding”. In addition, a spectrum of cell adhesion and junction-related processes exhibited significant shifts, including “cell junction disassembly” and “cell adhesion molecule binding”. Synaptic processes, represented by “synapse organization” and “synapse pruning”, also displayed enriched expression alterations. Finally, processes linked to differentiation and cellular development, such as “positive regulation of myeloid leukocyte differentiation”, “positive regulation of cell development”, and “positive regulation of osteoclast differentiation”, were markedly significant. A more complete overview of the top 20 enriched biological processes and molecular functions from the Gene Ontology database is shown in Fig. 2. Interestingly, the analysis of biological processes indicated overall a strong involvement of neuroinflammatory processes in THY-Tau22 mice.

#### Enrichment analysis of female-specific DEGs

**Fig. 3:**
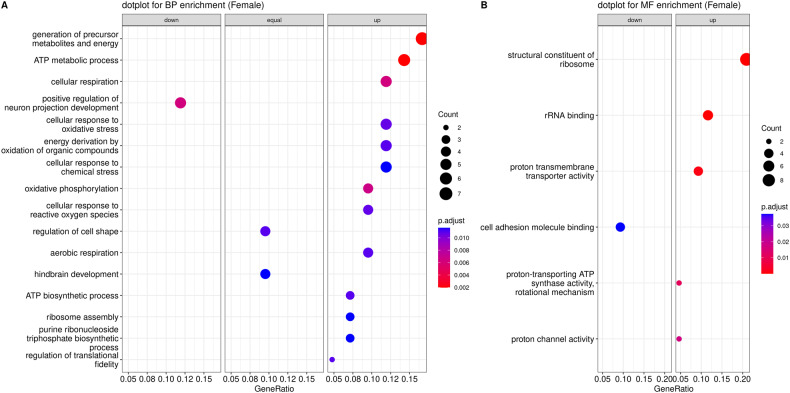
Dot plot visualization of female-specific changes in biological processes and molecular functions. Dot plot displaying the most significant Gene Ontology biological processes (BP) in (**A**), and molecular functions (MF) in (**B**), enriched in THY-Tau22-associated female-specific DEGs. The radius of the circles reflects the number of genes linked to specific GO terms, and the color gradient from red to blue reflects the adjusted *p*-value (p.adjust, see right side legend). The horizontal axis represents the gene ratio, indicating the extent of overlap between the input DEGs and the members of the pathway, relative to the overlap with all the members in the gene set collection. Wherever possible, each plot has been sub-categorized into three different pathway alteration trends, up, down, and equal, depending on whether the majority of DEGs displayed over-expression or under-expression, or whether an equal number of genes occurred in both categories.

In the analysis of female-specific DEGs, we observed a pronounced enrichment in cellular processes tied to energy metabolism. These included GO terms such as “generation of precursor metabolites and energy”, “ATP metabolic process”, “ATP biosynthetic process”, “cellular respiration”, “energy derivation by oxidation of organic compounds”, “aerobic respiration”, “oxidative phosphorylation”, and “proton-transporting ATP synthase activity, rotational mechanism”. In addition, pathways related to stress response, especially those linked to oxidative stress, manifested significant alterations in gene activity. These pathways included “cellular response to oxidative stress”, “cellular response to chemical stress”, “cellular response to reactive oxygen species”, and “response to reactive oxygen species”. Finally, pathways associated with ribosomes and protein translation also displayed enrichment in female-specific DEGs, highlighted by terms such as “ribosome assembly”, “structural constituent of ribosome”, “rRNA binding”, and “regulation of translational fidelity”. Figure 3 shows a dot plot of the most significant GO biological processes and molecular functions with a female-specific DEG enrichment. In summary, the analysis of biological processes suggests a prominent role of oxidative stress and associated response pathways in the expression changes observed in female THY-Tau22 mice.

#### Enrichment analysis of male-specific DEGs

**Fig. 4:**
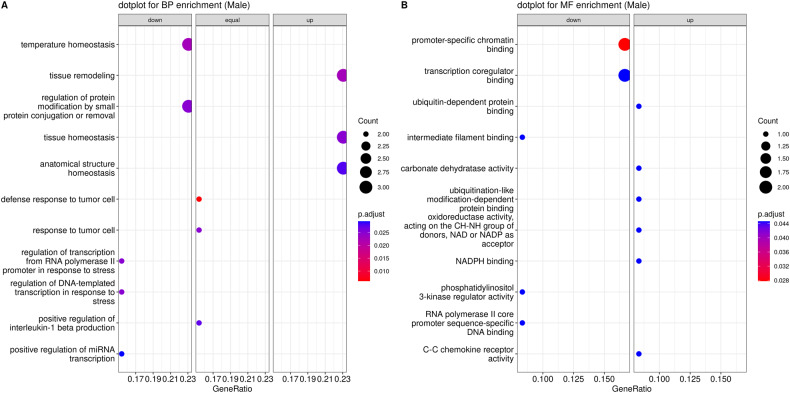
Dot plot visualization of male-specific changes in biological processes and molecular functions. Dot plot displaying the most significant Gene Ontology biological processes (BP) in (**A**), and molecular functions (MF) in (**B**), enriched in THY-Tau22-associated male-specific DEGs. The radius of the circles reflects the number of genes linked to specific GO terms, and the color gradient from red to blue reflects the adjusted *p*-value (p.adjust, see right side legend). The horizontal axis represents the gene ratio, indicating the extent of overlap between the input DEGs and the members of the pathway, relative to the overlap with all the members in the gene set collection. Wherever possible, each plot has been sub-categorized into three different pathway alteration trends, up, down, and equal, depending on whether the majority of DEGs displayed over-expression or under-expression, or whether an equal number of genes occurred in both categories.

An enrichment for DEGs with male-specific patterns was observed in particular for processes associated with interleukin-1 production, e.g., “positive regulation of interleukin-1 beta production”, “positive regulation of interleukin-1 production” and “regulation of interleukin-1 beta production”. In addition, there were significant shifts in pathways associated with miRNA transcription, such as “positive regulation of miRNA transcription”, “regulation of miRNA transcription”, and “miRNA transcription”. Stress response-related processes, specifically “regulation of transcription from RNA polymerase II in response to stress” and “regulation of DNA-templated transcription in response to stress”, were also prominently enriched in male-specific DEGs. Furthermore, pathways concerning tissue and anatomical structure remodeling and homeostasis, represented by “tissue remodeling”, “tissue homeostasis”, and “anatomical structure homeostasis”, were among the significant findings. Concludingly, molecular functions tied to chemokine binding and receptor activity, such as “C-C chemokine receptor activity”, “chemokine receptor activity”, “C-C chemokine binding”, and “chemokine binding”, exhibited marked alterations. Further details for the most significant GO terms are shown in Fig. 4.

In summary, both sex-neutral and sex-specific THY-Tau22-associated cellular process alterations were highlighted by the pathway analysis (see overview in Table 5 and a complete list in Suppl. Table 8). These included processes previously associated with AD, such as synapse organization and pruning, as well as pathways related to energy metabolism and oxidative stress response, mirroring our findings for the Tg2576 mouse model [17]. However, both these known AD-associated processes and newly identified significant pathway alterations, such as the male-specific alterations in miRNA transcription and regulation of interleukin 1 beta production, had not been linked to sex-specific differences in a tau model of AD before. To discern if these changes are unique to the THY-Tau22 model or represent broader patterns indicative of AD-like pathology, we compared them with data from the Tg2576 model [17] and *post mortem* brain samples from AD patients and controls [14] (detailed in the subsequent section).

### Comparison between transcriptomic changes in AD mouse models and human AD

**Fig. 5:**
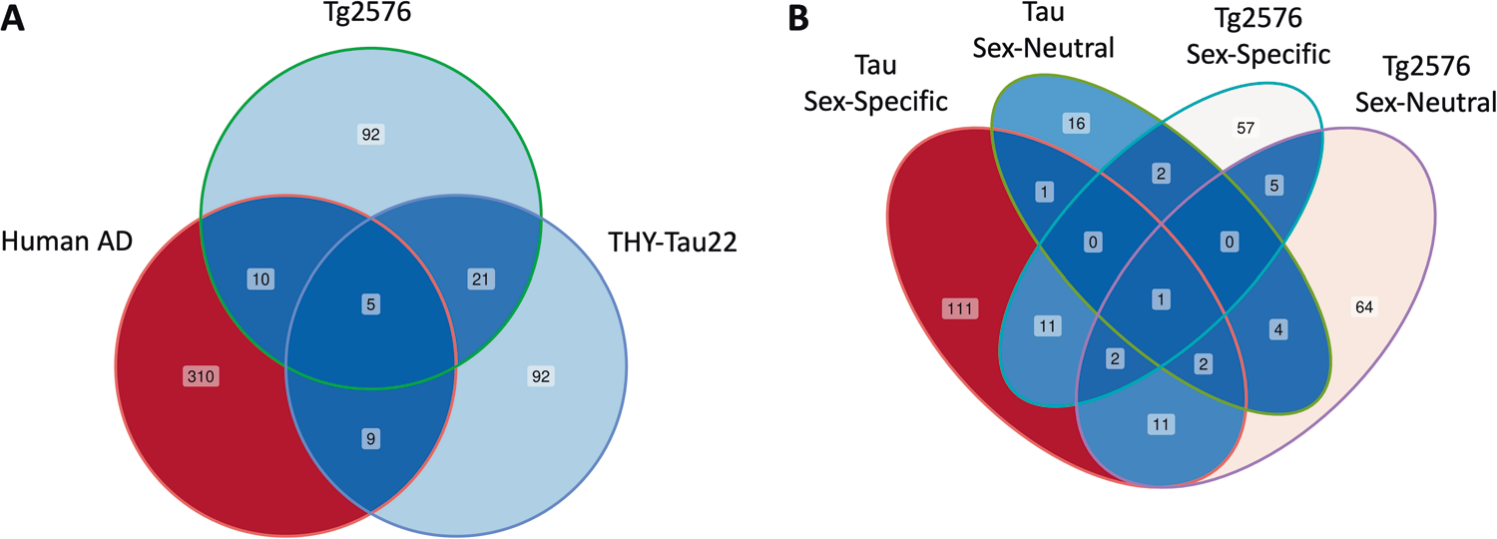
Venn diagrams of overlapping DEGs across different analyses. **A** Overlapping DEGs between the two studied mouse models of AD (THY-Tau22 and Tg2576) and the human AD brain transcriptomics dataset for 4 different cell types (microglial cells, oligodendrocytes, astrocytes, and neurons). **B** Overlapping DEGs between the two mouse models of AD (THY-Tau22 and Tg2576) for 6 cell types present in both datasets (microglial cells, oligodendrocytes, astrocytes, neurons, endothelial cells, and neuroblasts). Detailed statistics for these overlapping DEGs are provided in Suppl. Tab. 6.

We assessed the relevance of our findings for the THY-Tau22 model of AD by comparing them against our previously presented data for sex-dependent transcriptomic alterations in the cortex of the Tg2576 mouse model for AD compared to wild-type mice [17], as well as transcriptome changes in *post mortem* human brain tissues from the entorhinal cortex of AD compared to cognitively normal individuals [14]. Since all these studies used cortical single-cell RNA-seq (scRNA-seq) for gene expression profiling, we performed a cell type-specific comparison across these data to identify cross-species and cross-model shared significant changes for the four main cell types represented in all datasets (microglial cells, oligodendrocytes, astrocytes, and neurons). Overall, we found 5 distinct genes (*MBP*, *MALAT1*, *PLP1*, *HSP90AA1*, and *ACTB*) that consistently exhibited significance across all three datasets (Fig. 5A), including genes whose expression levels were altered in the same cell type across the different datasets. For instance, the *MBP* gene, encoding the myelin basic protein, emerged as an oligodendrocyte-specific DEG. It exhibited male-specific reduced expression in the Tg2576 model (logFC = −0.33, FDR = 1.8e−11) and human AD (logFC = −0.17, FDR = 6.1e−34). Conversely, in the THY-Tau22 AD mouse model, it showed a female-specific over-expression (logFC = 0.26, FDR = 5.2e−27). This suggests a nuanced, sex-dependent modulation of *MBP*, potentially contingent on whether the pathology is predominantly Tau or Abeta-driven. Similarly, a further gene encoding a myelin-associated glycoprotein, proteolipid protein 1 (*Plp1*), was identified as an oligodendrocyte-specific DEG. It consistently displayed decreased expression in AD males across Tg2576 (logFC = −0.32, FDR = 8.8e−142), THY-Tau22 (logFC = −0.03, FDR = 1.2e−03), and human AD samples (logFC = −0.10, FDR = 1.6e−12). While this under-expression was consistent between sexes in the THY-Tau22 model, a sex-dimorphic shift with elevated expression in females was evident in both the Tg2576 model and human AD. Notably, prior studies have reported a significant decline in PLP1 protein levels in the frontal cortex of AD brains relative to age-matched controls, as well as in vascular dementia [40]. However, these earlier studies did not account for potential sex-related disparities. The gene *HSP90AA1* (Heat Shock Protein 90 Alpha Family Class A Member 1) also emerged as a consistently differentially expressed gene in oligodendrocytes across all three datasets. Notably, this male-specific DEG displayed elevated expression in both THY-Tau22 mice (logFC = 0.19, FDR = 2.5E−09) and human AD patients (logFC = 0.41, FDR = 3.0E−156), while it showed reduced expression in Tg2576 males (logFC = −0.36, FDR = 3.5E−03). *HSP90AA1* encodes a molecular chaperone protein pivotal for ensuring the correct folding and stabilization of other proteins, especially those integral to cell signaling, cell cycle regulation, and stress response. The involvement of HSP90AA1 and its associated co-chaperones in various neurodegenerative conditions has been documented [41]. For instance, in the human microglia of the prefrontal cortex from AD patients, an over-expression of HSP90AA1 and other heat shock proteins has been observed, potentially in response to external stress [42].

In neurons, *ATP1B1* emerged as a female-specific DEG, displaying consistent under-expression in both THY-Tau22 mice (logFC = −0.51, FDR = 2.7e−06) and human AD (logFC = −0.37, FDR = 0.01), but was not detected in neurons in the Tg2576 dataset. This gene, encoding the Na/K-ATPase beta1-subunit, has already been shown to exhibit an under-expression in AD brains (logFC = −0.77, FDR = 0.003) and mRNA levels significantly negatively correlated with 5-hydroxymethylcytocine (5hmc) (R^2^ = −0.30, *P* = 0.008) [43], suggesting that it is tightly regulated at both transcriptomic and epigenetic levels. Two further cell types (endothelial cells and neuroblasts) were covered both in the THY-Tau22 and Tg2576 AD mouse models, but not in the human scRNA-seq dataset. We therefore investigated whether for these cell types, there are sex-dependent DEGs that share significance at least for the two mouse models (Suppl. Table 9). While in these cell types no genes with the same cell type annotations were found as shared DEGs, we identified *Tsc22d3* as a male-specific DEG, consistently under-expressed in male endothelial cells in the Tg2576 model and in male microglial cells in the THY-Tau22 model (Fig. 5B). *Tsc22d3* encodes a putative anti-inflammatory transcription factor and has been shown to express high mRNA levels in brain-infiltrating CD8 + T cells in AD mice [44]. In the same study, it was also reported to occur in an altered gene co-expression module identified in peripheral blood from AD patients, suggesting it may play a relevant role in neuroinflammatory processes associated with AD. Lastly, the gene *Gm42418*, which encodes a lncRNA of currently undetermined function, consistently exhibited reduced expression in both transgenic males and females within endothelial cells and oligodendrocytes across both mouse models.

### Cell type-agnostic gene regulatory network analysis

To gain a better understanding of the regulatory mechanisms underlying sex-dependent gene activity changes in the cortex of THY-Tau22 mice, we performed a differential gene regulatory network (GRN) analysis. First, a GRN analysis was conducted using a cell type-agnostic analysis of sex-specific DEGs, i.e., male- and female-specific transcriptomic alterations observed in the combination of cell types. Apart from identifying interpretable regulatory mechanisms, this also enabled us to determine relevant regulatory genes which may play key roles in modulating and controlling sex-dependent and disease-associated gene expression. Specifically, we applied a dedicated method for differential GRN inference [45] separately to the cell type-agnostic, sex-specific DEGs for males (14 DEGs) and females (52 DEGs) to reconstruct phenotype-specific gene networks.

#### Female-specific differential GRNs

**Fig. 6:**
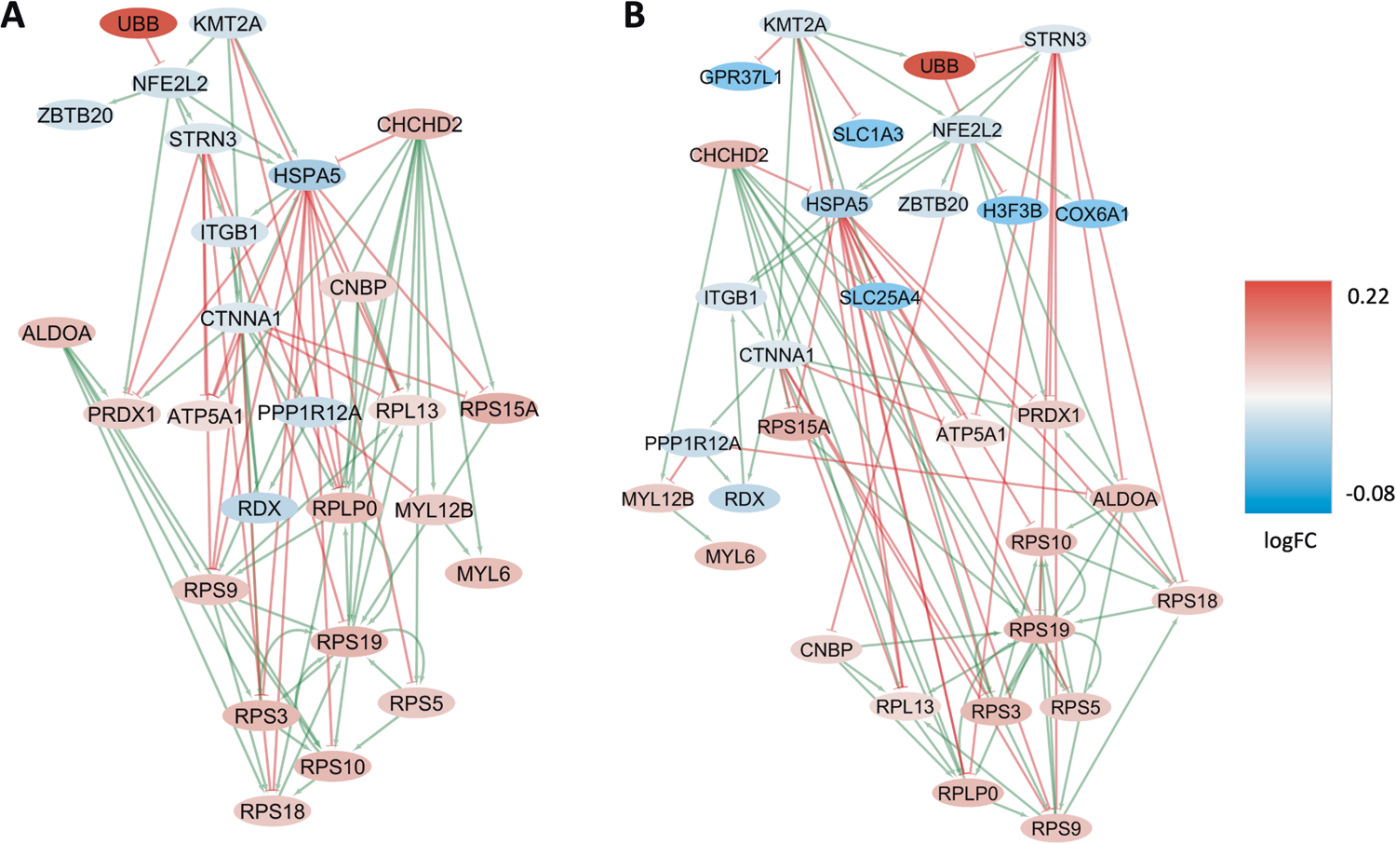
Condition-specific gene regulatory networks enriched in female-specific DEGs. Graph representation of the condition-specific GRNs for two genotypes, THY-Tau22 (**A**) and wild type (**B**), enriched in female-specific DEGs in the brain cortex tissue. These networks depict the upstream regulatory genes and transcription factors, along with their interactions with direct downstream target genes (arrows pointing downwards). The THY-Tau22 network (**A**) consists of 26 genes and 83 interactions, while the wild-type network (**B**) comprises 31 genes and 94 interactions. In the graph, green lines represent activating interactions, red lines represent inhibitory interactions, and the node colors indicate the gene expression log fold change in the respective phenotype, transitioning from red (over-expressed) to blue (under-expressed) as shown in the color legend on the right.

For the cell type-agnostic female-specific DEGs (FDR < 0.05, *N* = 52), we constructed two condition-specific GRNs representing the primary regulatory networks governing female-specific expression in the cortex of THY-Tau22 and wild-type mice, respectively. The THY-Tau22-specific network comprises 26 genes and 83 regulatory interactions (Fig. 6A), while the wild-type network covers 31 genes and 94 interactions (Fig. 6B). In these networks, several key regulatory genes and transcription factors were identified (Fig. 6A, B), including *Aldoa*, *Rps19*, and *Ubb*, all of which exhibit a shared significant decrease in female-specific expression in THY-Tau22 compared to wild type in the cortex. These genes regulate more than half of the genes in the GRN (Table 6), suggesting a pivotal role in mediating and controlling female-specific expression changes associated with THY-Tau22.

**Table 6:** Network perturbation analysis for sex-specific GRNs.

Female		Male	
Genes	Score	Genes	Score
*Ubb*	14	*Klf4*	2
*Rdx*	8	*Egr1*	2
*Aldoa, Rdx*	12	*Vim, Klf4*	2
*Ubb, Aldoa*	17		
*Ubb, Aldoa, Rps19*	20		
*Ubb, Aldoa, Rps19, Cnbp*	20		

Perturbation score analysis of the main regulatory genes identified in the network analysis of the THY-Tau22 GRN model, derived from the DEGs specific to females and males across all cell types (cell type-agnostic DEGs). The gene scores indicate how many downstream genes in the GRN can have their transgene-associated expression changes reverted back to the wild-type expression profile by modulating the activity of corresponding upstream regulators (scores are sorted first by increasing numbers of regulatory genes considered, and then by decreasing score, considering perturbagen combinations of up to 4 genes).

#### Male-specific differential GRNs

**Fig. 7:**
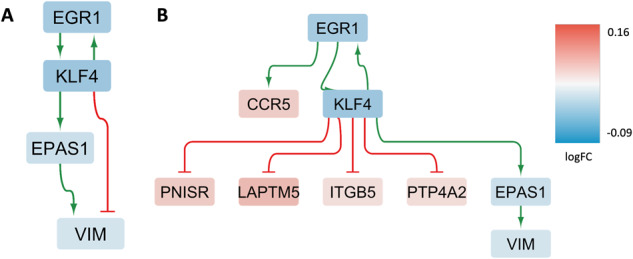
Condition-specific gene regulatory networks enriched in male-specific DEGs. Graph representation of the condition-specific Gene Regulatory Networks (GRNs) for two genotypes, THY-Tau22 (**A**) and wildtype (**B**), enriched in male-specific DEGs in cortical tissue. These networks depict the upstream regulatory genes and transcription factors, along with their interactions with direct downstream target genes. The THY-Tau22 network (**A**) consists of 4 genes and 5 interactions, while the wildtype network (**B**) comprises 9 genes and 9 interactions. In the graph, green lines represent activating interactions, red lines represent inhibitory interactions, and the node colors indicate the gene expression log fold change in the respective phenotype, transitioning from red (over-expressed) to blue (under-expressed).

Analogous to the female-specific GRN analysis, for the male-specific DEGs (FDR < 0.05, *N* = 14) two GRNs were reconstructed, representing the regulatory networks involved in controlling these genes in THY-Tau22 and wild-type mice. The THY-Tau22-specific network covers 4 genes and 5 regulatory interactions (Fig. 7A), and the wild-type network includes 9 genes and 9 interactions (Fig. 7B). As main regulatory genes identified in this analysis, the transcription factors *Klf4* and *Egr1* displayed significant male-specific decreased expression in THY-Tau22 mice vs. wild-type mice, and are involved in regulating almost half of the genes in the wild-type GRN (Table 6).

#### Cell type-specific sub-network enriched in female-specific DEGs (microglial cells)

**Fig. 8:**
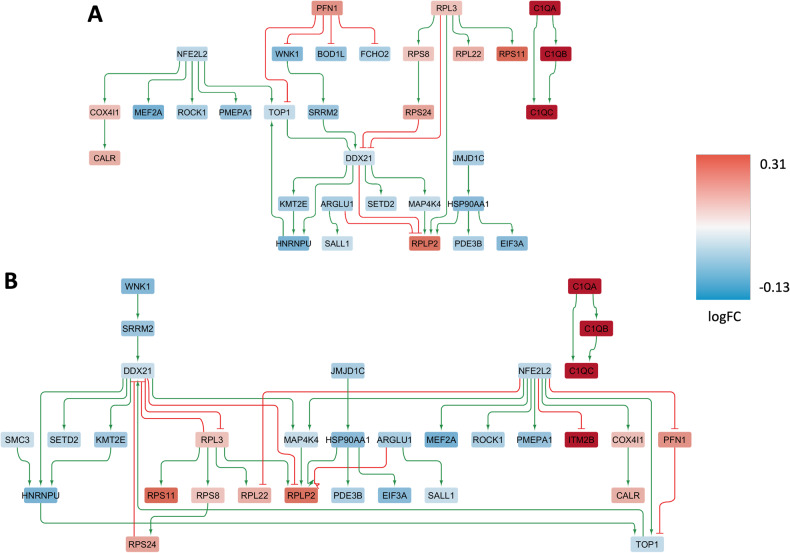
Condition-specific gene regulatory networks enriched in female-specific DEGs in brain microglial cells. Graph representation of the condition-specific GRNs for two genotypes, THY-Tau22 (**A**) and wild type (**B**), enriched with female-specific DEGs in brain microglial cells. These networks depict the upstream regulatory genes and transcription factors, along with their interactions with direct downstream target genes (arrows pointing downwards). The THY-Tau22 network (**A**) consists of 32 genes and 37 interactions, while the wild-type network (**B**) comprises 32 genes and 40 interactions. In the graph, green lines represent activating interactions, red lines represent inhibitory interactions, and the node colors indicate the gene expression log fold change in the respective phenotype, transitioning from red (over-expressed) to blue (under-expressed) as shown in the color legend on the right.

To study examples for sex-dependent cell type-specific sub-network alterations, we focused on cell types with high numbers of sex-specific DEGs, including microglial and endothelial cells. A set of 68 female-specific DEGs in microglial cells was identified and used as input to reconstruct the GRN. The condition-specific sub-networks generated for microglial cells include a THY-Tau22-specific network consisting of 32 genes and 37 regulatory interactions (Fig. 8A) and a wild-type network covering 32 genes and 40 interactions (Fig. 8B). Key upstream regulatory genes in the network include over-expressed *Rpl3* and under-expressed *Top1* and *Ddx21*, which displayed significant female-specific expression levels in microglial cells (Table 7).

**Table 7:** Network perturbation analysis for female-specific endothelial and microglial GRNs.

Female endothelial cells		Female microglial cells	
Genes	Score	Genes	Score
*Chchd2*	10	*Rpl3*	15
*Sfpq*	9	*Ddx21*	15
*Chchd2, Sfpq*	12	*Rpl3, Top1*	20
*Chchd2, Sfpq, Itga1*	11	*Rpl3, Hnrnpu*	20

Perturbation score analysis of the main regulatory genes identified in the network analysis of the THY-Tau22 GRN model, derived from the DEGs specific to females and males in cortical endothelial and microglial cells. The gene scores indicate how many downstream genes in the GRN can have their transgene-associated expression changes reverted back to the wild-type expression profile by modulating the activity of corresponding upstream regulators (scores are sorted first by increasing numbers of regulatory genes considered, and then by decreasing score, considering perturbagen combinations of up to 3 genes).

#### Cell type-specific sub-network enriched in female-specific DEGs (endothelial cells)

**Fig. 9:**
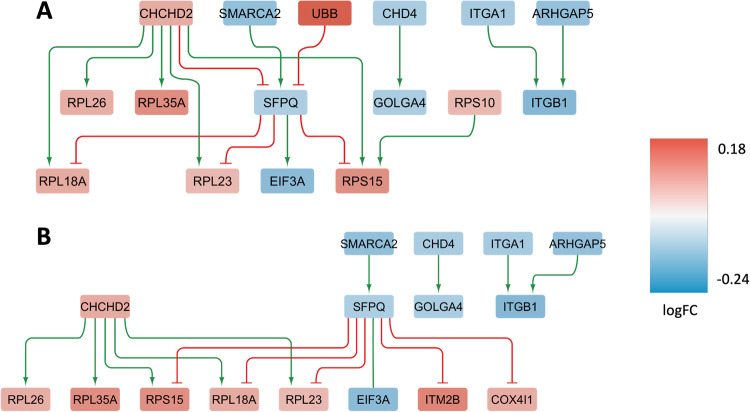
Condition-specific gene regulatory networks enriched in female-specific DEGs in brain endothelial cells. Graph representation of the condition-specific GRNs for two genotypes, THY-Tau22 (**A**) and wild type (**B**), enriched with female-specific DEGs in the brain endothelial cells. These networks depict the upstream regulatory genes and transcription factors, along with their interactions with direct downstream target genes (arrows pointing downwards). The THY-Tau22 network (**A**) consists of 16 genes and 16 interactions, while the wild-type network (**B**) comprises 16 genes and 15 interactions. In the graph, green lines represent activating interactions, red lines represent inhibitory interactions, and the node colors indicate the gene expression log fold change in the respective phenotype, transitioning from red (over-expressed) to blue (under-expressed) as shown in the color legend on the right.

In endothelial cells, the construction of cell type-specific sub-networks using the 41 female-specific DEGs identified 16 genes and 16 regulatory interactions in the THY-Tau22-specific network (Fig. 9A), while the corresponding wild-type network included 16 genes and 15 interactions (Fig. 9B). Notably, two upstream regulatory genes, *Chchd2* and *Sfpq*, exhibited significant female-specific differential expression in THY-Tau22 mice vs. controls (Fig. 9A, B).

## Discussion

Our analyses of the THY-Tau22 model of AD have revealed statistically significant cell type-specific and cell type-agnostic changes at an early stage of disease initiation (7 months), using stringent significance cut-offs after multiple hypothesis testing adjustments (FDR < 0.05). While many genes displayed a sex-neutral differential expression, we also identified significant male- and female-specific DEGs across multiple cell types as well as sex-dimorphic DEGs with opposite directions of the change. When we further explored potential coordination patterns of these sex-dependent DEGs in cellular processes and networks, a number of pathways previously implicated in AD were significantly enriched in male- or female-specific DEGs, including pathway changes shared with the previously studied Tg2576 model of AD and changes seen in human *post-mortem* cortex samples from AD patients. To gain a better mechanistic understanding of these changes, the GRN analyses highlighted upstream regulatory genes that may play a central role in controlling the observed downstream expression changes. One of the most consistent findings across the THY-Tau22 and Tg2576 mouse models and human AD patient samples was a sex-dependent dysregulation of myelin-associated genes and proteins in oligodendrocytes, including *Mbp* and *Plp1*. The alterations in these genes, which are involved in myelin integrity and repair, suggest that disruption of myelin plasticity may be an early event in AD progression. Given previous studies showing beneficial effects of *Mbp* overexpression on axonal regeneration in a spinal cord injury model [46] and reduced amyloid pathology in an AD model [47], this protein may warrant further investigation as a potential therapeutic target to promote neuroprotection in AD. Below, we discuss each category of observed cell type-agnostic and cell type-specific changes in detail in the context of the previous literature on AD.

### Sex-Neutral DEGs

Overall, for the 10 different cell types studied, we identified 45 DEGs with a shared direction of the change across the sexes. Sex-neutral alterations were observed in particular in oligodendrocytes (14 DEGs), including important regulatory genes such as *Malat1*, *Car2*, *Neat1*, and *Plp1* (see Table 1). Oligodendrocytes have important supporting functions for neurons and play a significant role in the formation of myelin, which acts as an insulator of axonal segments [48, 49]. Previous studies have proposed that the initiation of Alzheimer’s disease (AD) pathology may be rooted in a compromised repair mechanism of oligodendrocyte precursor cells (OPCs), ultimately resulting in the breakdown of myelin [33, 50]. Our findings provide additional support to the concept that transcriptional changes in oligodendrocytes, occurring significantly prior to the manifestation of AD-like symptoms [51], are discernible in the brains of a relevant mouse model. Besides oligodendrocytes, the most pronounced sex-neutral alterations were detected in microglial cells (10 DEGs), including genes with well-known AD-associations, such as *Apoe*, *Lgmn*, and *Actb* (see Table 1). *Apoe* is a cholesterol carrier that supports lipid transport and injury repair in the brain, and the E4 variant of the gene is known as the most influential risk factor for late-onset AD [52, 53]. For the legumain gene (*Lgmn*), functional studies have demonstrated that it cleaves both a precursor of Abeta and tau, and alterations in its protein levels have been associated with the formation of neurofibrillary tangles and senile plaques as key hallmarks of AD [54]. Finally, the gene *Actb*, which encodes the important cytoskeletal protein beta actin, is known to be impacted by cytoskeletal dysregulation in AD. In particular, so-called Hirano bodies, paracrystalline intracellular inclusions containing actin and actin-binding proteins, are a prominent characteristic of AD brains [55]. Overall, these results indicate oligodendrocytes and microglial cells as key cell types influenced by significant sex-neutral transcriptomic alterations in AD-like pathologies. Apart from the observed cell type-specific changes, some genes also showed consistent sex-neutral alterations across multiple cell types. One of the genes with the most pronounced alterations in this category is *Ttr* (Transthyretin), with altered expression in transgenic mice in 8 out of 10 different brain cell types (see Table 1). *Ttr*, a carrier protein for retinol and thyroxine in cerebrospinal fluid (CSF) and plasma, has well-established protective roles in AD, and as a main Abeta binding protein in the CSF, it is thought to prevent its aggregation and toxicity [56, 57]. Furthermore, overexpressing human wild-type *TTR* in an AD mouse model has been shown to reduce neuropathology and Aβ deposition [58]. Thus, while significant cell type-specific changes occur in the THY-Tau22, some of the potential candidate drug targets also include genes with a largely cell type independent dysregulation.

### Sex-specific DEGs

As a general observation, we detected more female-specific than male-specific changes in the THY-Tau22 mouse model. While this matches with reports for scRNA-seq changes in human AD [15], we had previously found an opposite pattern of predominant male-specific alterations for an Abeta mouse model of AD [17]. This may indicate a stronger association of female-specific alterations in AD with Tau pathology, in line with prior studies showing that female patients with AD tend to have more pathological tau in the brain and CSF than men [59]. However, while further studies will be required to confirm this generic disparity in sex-dependent AD-like pathology in tau- and Abeta-based models of AD, we also observe shared sex-specific alterations between the THY-Tau22 model and the Tg2576 model (see details below), suggesting that there is also at least a partial convergence between sex-related downstream pathology in both models.

Overall, across 10 different cell types, we identified 169 female-specific and 9 male-specific DEGs, with microglial cells having the highest count of sex-specific DEGs (68 female-specific DEGs, and 4 male-specific DEGs). Some of these DEGs with pronounced alterations include complement system genes (*C1qa*, *C1qb*, and *C1qc*) as well as transcriptional regulators, such as *Klf6*, *Trem2*, *Cd81*, and *Cd68*. The complement system genes are well-known markers of microglial cells, and their over-expression in females (FDR < 3.1E−80) may indicate over-activation of these cells in the AD phenotype. Alterations in their protein levels have been observed in the earliest stages of amyloid deposition in AD, and activation of these proteins has been detected concurrent with the clinical onset of dementia [60]. Next, the gene *Trem2* encodes the protein *“*triggering receptor expressed on myeloid cells-2”, a receptor of the innate immune system expressed in several brain cell types [61]. Trem2 is involved in microglial functions as well as immune system modulation and harbors genetic variants found to increase risk for late-onset AD (LOAD) [62, 63]. Furthermore, sex-specific changes in immune system modulators, such as *Cd81* and *Cd61*, have previously already been observed in microglial cells [64] and in the sera of AD patients [65]. Taken together, these results suggest that AD-like tau pathology is strongly associated with sex-specific changes across multiple cell types, with microglial changes being particularly pronounced.

### Sex-dimorphic DEGs

While most genes with sex-dependent changes identified in our analyses displayed pronounced alterations only in one of the sexes, we also identified a few genes with significant sex-dimorphic patterns, i.e., significant changes in both sexes, but with an opposite direction of the change. These sex-dimorphic DEGs were observed in OPCs (*Ttr, Malat1, Slc1a2, mt-Rnr1*, and *mt-Rnr2*), astrocytes (*Aldoc*), mural cells (*Atp1a2*, *Mgp*, and *Ptn*), and ependymal cells (*Nnat*, see Table 3). *Malat1*, *mt-Rnr1, mtRnr2, Aldoc*, *Slc1a2*, *Mgp*, and *Nnat* all displayed significantly elevated levels in THY-Tau22 females vs. wild-type females, and an opposite pattern was observed in males.

Interestingly, in our previous study of the Tg2576 mouse model of AD [17], we had already observed the same sex-dimorphic pattern for *mt-Rnr2* in neuronal cells from the neocortex, with an increase in the transgenic females and a decrease in the transgenic males. The gene *mt-Rnr2* has been reported to perform a dual function, encoding for a 16 S mitochondrial subunit ribosomal RNA and a micropeptide known as humanin [66]. Humanin exhibits a variety of neuroprotective effects, for instance, both secreted and synthetic humanin peptides have been shown to protect neuronal cells from damage caused by accumulation of the 42-amino acid variant of Abeta (Aβ42) in the brain [67], and protective effects were described for multiple in vitro and in vivo models of neurodegenerative disorders [66, 68–71]. The proposed mechanisms are the competitive inhibition of the access of the receptor FPR1 to Aβ42 and antagonizing the impact of Aβ42 on mononuclear phagocytes [67]. In addition, a significant reduction in humanin protein plasma levels with increasing age has been observed across both mice and humans, suggesting a possible association with aging processes [70, 72]. Furthermore, in human AD, humanin protein levels are significantly decreased compared to controls in the CSF, and were associated with low mitochondrial DNA copy numbers, indicating a relation with mitochondrial dysfunction in AD [72]. Since humanin displays consistent sex-dependent changes in human AD and two mouse models and has neuroprotective properties, it may serve as a target for the preclinical development and testing of pharmacological strategies to promote neuroprotection in AD. While no other gene apart from *mt-Rnr2* displayed shared sex-dimorphic alterations across human AD and the two mouse models, we observed that *Malat1* displayed significant sex-dependent changes also in the Tg2756 model. In contrast to the male-specific decrease and female-specific increase of *Malat1* observed in THY-Tau22 mice, the gene displayed significant decreased levels in both sexes in endothelial cells in the Tg2576 models, but with a lower log fold change in males (−0.56) than females (−0.34). This suggests that *Malat1* is modulated by sex-dependent processes in both animal models, but with significant variation in the type and magnitude of the effect across models. In summary, multiple significant sex-dimorphic DEGs were identified in the THY-Tau22 model, including genes with well-established functional links to AD and genes with sex-dependent alterations observed also in an independent AD mouse model and in human AD.

### Comparison of sex-neutral and sex-dependent transcriptomic changes across model systems and human AD

In order to assess which sex-neutral and sex-dependent transcriptomic changes in the THY-Tau22 model are specific to this model and which changes are shared with an Abeta-model of AD and with human AD, we systematically compared the identified cell type-specific DEGs against those previously detected in scRNA-seq data from the Tg2576 mouse model of Abeta pathology [17] and from *post mortem* cortex samples of human AD patients [14]. One of the genes with shared significance in oligodendrocytes is *MBP*, a commonly used oligodendrocyte marker, which showed female-specific increased expression levels in THY-Tau22 mice (logFC = 0.26, FDR = 5.2e−27) and a male-specific decreased expression in Tg2576 mice (logFC = −0.33, FDR = 1.8e−11) and in human AD (logFC = −0.17, FDR = 6.1e−34, Supplementary Table 10). *MBP* encodes myelin basic protein, an important component of the myelin sheath, the insulating layer formed around nerves to facilitate fast and efficient transmission of electrical impulses. Myelin dysfunction has been linked to neuronal impairment and cognitive decline, and neuroimaging and *post mortem* human brain studies revealed that myelin deterioration is correlated with the presence of Abeta (Aβ) plaques and tau hyperphosphorylation [73, 74]. Furthermore, *MBP* has been reported to associate with amyloid plaques, AβPP, and Aβ1-42 in the cortex from AD patients [75], and a decrease of *MBP* expression was observed in a further AD mouse model, 6-month-old APP/PS1 mice, compared to wild-type mice [76]. Multiple prior studies also suggest that MBP might serve as a potential target for neuroprotection. For example, in vivo overexpression of MBP was shown to significantly enhance the locomotor recovery and axonal regeneration in post-spinal cord injury mice [46]. The same study also suggested that myelin promotes axonal outgrowth from neural progenitor cells in an MBP-dependent manner. Moreover, an independent study showed that oral delivery of bioencapsulated MBP reduces Aβ42 aggregates in the hippocampus and cortex brain regions of the 3xTg mouse model of AD [47]. In the context of other neurodegenerative disorders, treatment strategies involving MBP-based peptides are being investigated in clinical trials for multiple sclerosis [77] (NCT02903537). Finally, although sex-dependencies in *MBP* levels have not been discussed in the context of AD before, hormone-mediated lower *MBP* expression levels in males than females have already been described for the orbital frontal cortex in rats [60, 78]. Interestingly, a further myelin-associated glycoprotein, proteolipid protein 1 (*Plp1*, also known as lipophilin), displayed consistent oligo-dendrocyte-specific under-expression in males for THY-Tau22 mice (logFC = −0.03, FDR = 1.2e−03), Tg2576 mice (logFC = −0.32, FDR = 8.8e−142), and in human AD (logFC = −0.10, FDR = 1.6e−12). These results are also supported by an independent study reporting reduced PLP1 protein levels in the frontal cortex of AD brains compared to age-matched controls, and in particular for patients with vascular dementia [40]. Furthermore, prior experimental evidence suggests that PLP1 engages in protein-protein interactions with MBP [79, 80] indicating their involvement in shared biological pathways. Taken together, these observations demonstrate consistent alterations in myelin-associated glycoproteins in oligodendrocytes in both human Alzheimer’s disease (AD) and AD mouse models. Given the beneficial effects previously observed for MBP overexpression in an AD model system, this protein may hold promise as a potential drug target for promoting neuroprotection in AD.

### Network analyses

#### Female-specific network (cell type-agnostic analysis)

The cell type-agnostic gene regulatory network (GRN) analysis of global female DEGs identified the gene *Aldoa* as a relevant upstream regulator in the network, among others (Fig. 6A and Table 6, left side). *Aldoa* exhibits a marked increase in transgene-associated expression (FDR = 1.9E24). Together with other regulatory genes (see Table 6), it has the potential to modulate the gene expression state of almost half the genes in the female GRN. *Aldoa* encodes the aldolase A protein, closely related to aldolase C (discussed above among the top significant genes with sex-dimorphic changes). It has been implicated in AD by multiple studies. For example, sequence variations in the human *ALDOA* gene have been associated with AD, and the expression of both the gene and corresponding protein have been proposed as a biomarker for AD [81, 82]. Furthermore, altered levels of the human gene product, aldolase A, which serves a regulator of glycolysis, may contribute to glycolytic abnormalities in AD patients [83].

#### Male-specific network analysis (cell type-agnostic analysis)

The cell type-agnostic male-specific GRN analysis and network perturbation scoring highlighted the roles of two upstream regulator genes, *Egr1* and *Klf4*, in controlling the expression of many downstream genes in this GRN (see Fig. 7 and Tab. 6, right side). Interestingly, these two genes had already been identified as key regulators and sex-dimorphic DEGs in our previous study of the Tg2576 mouse model of AD [17]. Although the sex-dependent dysregulation patterns vary across the two mouse models, the shared upstream regulatory control of relevant network alterations across the two mouse models suggests that these genes have important roles in mediating sex-dependent expression changes in AD-like pathologies.

##### Key regulatory gene 1—Egr1

The transcription factor *Egr1* (Early growth response-1), which has significantly decreased expression levels (FDR = 1.5E12) in THY-Tau22 males, is a so-called “immediate early gene”, that is rapidly transcribed in response to cellular stimuli. In our previous scRNA-seq analysis of the Tg2576 mouse model of AD, we had already observed multiple sex-dependent significant alterations in *Egr1* across different cell types, including a sex-dimorphic change in astrocytes (logFC in males: − 0.31, FDR: 2.82E-07; logFC in females: 0.53, FDR: 0.00016) and a male-specific decrease in endothelial cells (logFC: −0.26, FDR: 0.0012) [17]. Furthermore, our previous literature review of known *Egr1* functions and prior implications in AD indicated that the corresponding protein might serve as a candidate drug target for neuroprotective therapies: In the 3xTg mouse model of AD, inhibiting *Egr1* expression in the hippocampus was associated with several positive outcomes, such as diminished Abeta pathology, improved cognitive performance, and reduced tau phosphorylation [84]. Moreover, in both human biospecimens and AD mouse models *Egr1* was found to upregulate the enzyme acetylcholine-esterase (AchE) [85]. Consequently, inhibiting *Egr1* could offer a strategy to counteract the pathological depletion of acetylcholine in AD. Given that human *EGR1* stimulates presenilin-2 in neuronal cells, inhibiting *Egr1/EGR1* might also inhibit the amyloidogenic processing of APP [86]. However, as we noted in our previous study on the Tg2756 model, *Egr1/EGR1* also plays an important role in memory formation, and a too strong inhibition of this target protein could be detrimental. Thus, further studies will be required both to confirm the suitability of *Egr1/EGR1* as an AD drug target and to determine a suitable level of partial inhibition to achieve a pharmacological effect within the therapeutic window.

##### Key regulatory gene 2—Klf4

Apart from *Egr1*, another high-ranking gene in the perturbation analysis of the male-specific GRN was *Klf4*, which belongs to the Kruppel family of transcription factors. It exhibits a significant decrease in expression (FDR = 4.1E−24) within the THY-Tau22 samples. This aligns with our prior cell type-agnostic GRN analysis of the Tg2576 mouse model, where Klf4 was pinpointed as a central regulator controlling the GRN linking male-specific DEGs (see Fig. 8 in the original publication on the Tg2576 model [17]). *Klf4* and *Egr1* directly regulate each other in a feedback loop of the male-specific GRN (see Fig. 7), and are therefore involved in the control of the same downstream DEGs. Interestingly, *Klf4* also regulates the gene *Malat1*, which displayed significant sex-associated changes across multiple cell types in both the THY-Tau22 model (see Tables 1–3) and the Tg2576 model [17]. Previous experimental research has highlighted the role of *Klf4* in activating *Malat1* as part of protective mechanisms in response to ischemic stroke [87]. In addition, *Klf4* has been demonstrated to alleviate vascular damage following cerebral ischemic stroke by modulating the expression of tight junction proteins [88]. This matches with the significant pathway alterations observed for cell adhesion processes for both the Tg2576 model and the THY-Tau22 model. However, *Klf4* is a pleiotropic gene, with several other potential AD-relevant functional annotations. This includes a reported involvement in axon regeneration, apoptotic processes, and neuroinflammation, among others [89]. Therefore, further studies will be needed to elucidate the more precise role of *Klf4* expression alterations in AD-like pathologies.

#### Cell type-specific network analyses

As highlighted earlier, microglial and endothelial cell types exhibited the strongest sex-specific transcriptomic alterations, with 68 and 41 female-specific DEGs, respectively. The reconstructed sex-specific GRNs for these cell types revealed coordinated sub-network activity modulations (see Figs. 8 and 9). These are mainly controlled by two genes identified as potential master regulators in the network perturbation analysis, *Rpl3* (FDR = 2.8E−05; controlling 15 downstream target genes or 63% of the network nodes) and *Chchd2* (FDR = 0.006; controlling 10 downstream genes or 47% of the GRN nodes; see Table 7). Both genes displayed a significant female-specific increased expression in the respective cell types. Interestingly, in a study on blood biomarkers for early diagnosis of AD, using RNA-seq samples from 271 AD patients, 91 cognitively normal adults, and 248 individuals with mild cognitive impairment (MCI), the human gene *RPL3* was under-expressed in AD compared to cognitively normal controls along with other ribosomal genes [73]. *RPL3* has been linked to the modulation of mitochondrial activity through a mechanism in which the relative expression of *RPL3* and its paralog *RPL3L* alter interactions between ribosomes and mitochondria [74], suggesting a possible relation between *RPL3* dysregulation and mitochondrial dysfunction as a hallmark of AD. The primary regulator pinpointed in the endothelial GRN, *Chchd2*, is well-recognized for its mitochondrial localization, particularly in overseeing electron flow within the electron transport chain [90]. Recent findings have revealed two missense variants in this gene associated with AD risk in the Chinese Han demographic [91]. In addition, Chchd2 has been associated with potential causative sequence variants for another neurodegenerative disorder, Parkinson’s disease, in both Asian [92] and Caucasian [93] cohorts. While the exact functional relationships between *Chchd2* and neurodegeneration remain elusive, the identification of several potential pathogenic variants in this gene’s coding region in both AD and Frontotemporal Dementia [94] underscores its potential significance, suggesting that it may warrant further investigation in functional genomic studies.

Overall, the GRN analyses revealed a variety of sex-dependent, coordinated alterations in the transgenic mice. The main upstream regulator genes controlling these sub-networks, such as *Egr1*, and *Klf4*, include genes with consistent alterations observed also in the Tg2576 mouse model of AD. Notably, multiple of the regulator genes scoring highly in the network perturbation analysis have functional associations associated with mitochondrial activity (*Rpl3*, *Chchd2*), or carry variants with previously proposed pathogenic roles in AD (*Chchd2*). These genes will require further investigation and validation in AD model systems to confirm their functional relevance in the disease context and elucidate the associated mechanisms.

In conclusion, our scRNA-seq analysis of the THY-Tau22 mouse model of AD revealed significant cell type-specific and cell type-agnostic gene expression changes at an early stage of the disease, including both sex-neutral and sex-dependent alterations. The more dominant changes observed in females are consistent with similar findings for scRNA-seq changes in human AD, but contrast with the opposite pattern of predominantly male-specific changes seen in the Tg2576 Abeta mouse model. However, the affected genes and pathways in THY-Tau22 mice showed significant overlap with transcriptomic dysregulations previously observed in the Tg2576 model and human AD patient samples. Most notably, we uncovered consistent sex-dependent dysregulations of myelin-associated genes such as *Mbp* and *Plp1* in oligodendrocytes in all three datasets. Given previous evidence for beneficial effects of *Mbp* overexpression on axonal regeneration in spinal cord injury models and reduced amyloid pathology in AD models, this protein warrants exploration as a potential target for promoting neuroprotection. More broadly, our findings provide insight into early sex-related molecular mechanisms that influence AD susceptibility and progression at the single-cell level. This may pave the way for future studies to validate the functional relevance of the identified candidate genes in cellular and animal models and to assess their potential for the development of more personalized, sex-stratified diagnostics and therapies for AD.

## Materials and methods

### Transgenic THY-Tau22 mice

The AD mouse model used was originally described by Schindowski et al. [18]. It is based on transgenic overexpression of human 4-repeat, doubly mutated tau (G272V and P301S), driven by the Thy1.2 promoter, and named THY-Tau22 mice. Their AD-like phenotype is the age-dependent appearance of cognitive deficits, synaptic dysfunction, astrogliosis, and tau aggregation and inclusion formation, without neuromotor dysfunction, unlike many other similar models [18, 21]. We picked 7-month-old mice that represent an early time point of pathological development in the cortex of THY-Tau22 mice as we were interested in early stages of the disease process, thus increasing the likelihood of uncovering novel biomarkers or targets that ultimately enable early intervention in humans.

### Mouse husbandry, genotyping, and tissue work-up

THY-Tau22 breeders obtained from the University of Lille, France, were rederived and bred in Luxembourg by crossing heterozygous male breeders with commercially obtained C57Bl6/J females (RRID:MGI:3028467). The mice were kept in specialized facilities maintained under specific pathogen-free conditions. They were provided with unrestricted access to standard mouse food (Ssniff, # V 1534-300) and water at all times. Offspring was let age until 28 weeks. Five mice of each sex (male and female) per genotype (transgenic and wild type) were selected for this study (N = 20). This sample size was determined using a dedicated power calculation, ensuring a minimum power of 0.8 and an accepted type I error probability of 0.05 in a two-tailed T-test for a minimum effect size above 2 (calculated using the software G*Power 3.1). As this study examines variations across genotypes and sexes without incorporating any interventions, no blinding or randomization was used. Genotyping was done by PCR on DNA Proteinase K digested tail biopsies (Quantabio) per manufacturer’s instructions. We utilized a genotyping kit (Kapabiosystems, KR0385-v2.13) following previously established protocols [95]. A 250-bp fragment of the Microtubule-Associated-Protein Tau (MAPT) gene was amplified with a forward primer (5’ATGGCTGAGCCCCGCCAGGAG-3’) and a reverse primer (5’-TGGAGGTTCACCAGAGCTGGG-3’) as described [18], and, after separation of the PCR mix on an 1.5% agarose gel and incubation with gel red, viewed under ultraviolet light.

At the age of 7 months, the mice were humanely euthanized using deep anesthesia (ketamine 150 mg/kg, medetomidine 1 mg/kg, administered intraperitoneally) and subsequently transcardially perfused with phosphate-buffered saline (PBS) to eliminate blood residues. The brains were promptly extracted from the skulls. One hemisphere was preserved for histological examination (as described below), while the other hemisphere was dissected to isolate specific brain regions. The freshly dissected cortex was used for single-cell DropSeq analysis.

All mouse experiments were conducted in accordance with the European FELASA guidelines for animal experimentation and received approval from the local institutional Animal Experimentation Ethics Committee. Furthermore, experiments were overseen and approved by the relevant government agencies in Luxembourg, including the Ministry of Agriculture and the Ministry of Health.

### Thioflavin-S staining of intraneuronal tau inclusions

To visualize pathological tau inclusions independently of the state of hyperphosphorylation, we used a thioflavin-S (Thio-S) staining [96]. Two 50 µM thick brain sections, generated with a Leica VT1000 vibratome, from each THY-Tau22 mouse were mounted on superfrost plus glass slides and dried overnight before being stained with Thioflavin S. Two wild-type littermate mice of each sex were used as negative controls. Sections were treated with 10% formalin (VWR, 11699404) for 10 min and washed twice 5 min with PBS (Merck, 524650) before being incubated in 0.25% potassium metabisulfite (Sigma, 60458). After being washed in PBS for 5 min twice, sections were incubated in 2% potassium metabisulfite (Sigma, 60458) and 1% oxalate (Sigma, 75668) for 3 min (until white), washed twice in water and stained in 0.015% Thioflavin S (Sigma, T1892) in 50% ethanol (VWR 85933.290) for 10 min. Stainings were cleared in 50% Ethanol for 5 min and rinsed for 5 min in water. Sections were dried and coverslipped using Fluoromount G. 24 h later, they were observed with the AxioImager1 (Zeiss) with settings for Alexa 488 detection at ×20 magnification, and Thioflavin S positive cells were counted manually with a cell counter in the overall cortex and hippocampal CA1. For each mouse and each brain region, the number of cells counted was averaged. Results were statistically analyzed using the Student’s T-test, after a normality check, by applying the Graphpad Prism 9 software (RRID:SCR_002798).

### RNA extraction and sequencing

RNA was extracted from the cortex using the RNeasy Universal Kit (Qiagen) as previously described [97]. RNA samples were considered of acceptable quality when their RNA integrity value exceeded 8.5, and their absorbance ratios at 260/230 and 260/280 were equal to or greater than 1.8 and 2, respectively. These high-quality RNA samples were subsequently preserved at −80 °C until they were used for analysis. For single-cell gene expression profiling, freshly dissected mouse cortex was dissociated using Adult - Brain Dissociation (Miltenyi Biotec). The procedures to obtain material for single-cell RNA-seq and sequencing (microfluidics, single-cell droplet encapsulation, next-generation sequencing library preparation, bar-coded sequencing) were performed as previously described [17].

### Single-cell data quality filtering and pre-processing

Analyses of the single-cell transcriptomic data were performed using the Seurat software package (version 4.3.0, RRID:SCR_007322) in the R programming language (version 4.2.2, RRID:SCR_001905) [98]. The data underwent a dedicated processing pipeline, covering multiple quality control, cell selection, and filtration steps, as previously described [17]. Standard quality checks in the Seurat software did not highlight any problematic sample, and all input samples were consequently included in the subsequent analytical stages. To ensure data reliability, cells of substandard quality were excluded if unique feature counts were greater than 2500 or less than 200 to address potential issues related to cell duplication or sequencing of empty droplets. In addition, cells with mitochondrial gene counts greater than 5% were also filtered out, as elevated expression levels of mitochondrial genes may indicate apoptotic or deteriorating cells. The data were then normalized and scaled using a global scaling normalization implemented in the function *SCTransform* of the Seurat package [99]. Furthermore, to remove uninformative features, genes with low variation were filtered out using the *FindVariableFeatures* function and retaining only the top 2500 genes with the highest variance in the selection. Finally, we introduced a linear scaling transformation using the *ScaleData* function with default parameters. After these pre-processing and quality control analyses, the final data set covered a total of 44,910 single cells as input for the subsequent analyses.

### Single-cell clustering and cell type annotation

To identify, visualize, and annotate clusters of cells representing different cell types in the pre-processed data, we mapped the data to a lower dimension using a principal component analysis (PCA) and the Uniform Manifold Approximation and Projection (UMAP) method [25]. A suitable number of principal components (PCs) for the subsequent clustering analysis was determined using the Elbow method from the Seurat package and the Cumulative Sums (*cumsum*) function from the R package *base*. Briefly, cumulative percentages were calculated for each PC to identify the optimal threshold value where the percentage change in variance between successive PCs falls below 0.1%. In this case, the first 15 PCs were selected. Next, we applied a shared nearest neighbor (SNN) graph-based clustering to robustly assign individual cells to homogeneous groups with similar gene expression profiles [24] (see Fig. 1). The number of clusters was chosen to maximize the average *Silhouette Width* cluster validity index [100], as implemented in the R package *cluster* (version 2.1.4, RRID:SCR_013505), and the results were visualized using the R package *ggplot2* (version 3.4.2, RRID:SCR_014601). The final clustering was determined using the *FindNeighbors* and *FindClusters* functions in Seurat. To annotate the resulting clusters with corresponding cell types, we applied the method *ScType* [26]. This approach uses combinations of known marker genes for each cell type, derived from prior benchmark datasets, to assign cell type identities to the clusters according to the gene expression profiles of these known markers. Here, we obtained the list of cell marker genes from the public *Cell Marker* database (RRID:SCR_018503) [27]. Finally, the assigned cell type identities were validated by comparing the top differentially expressed genes between each labeled cluster and all other clusters against the established marker genes reported in the *Panglao* database [101] (RRID:SCR_022580). Overall, this clustering and annotation workflow enabled the cell type annotation of clusters with high confidence, facilitating subsequent analyses and data interpretation.

### Sex-dependent differential gene expression analysis

Differentially expressed genes (DEGs) between the sample groups of interest were scored by fitting a generalized linear model (GLM) using the Poisson distribution and the *FindMarkers* function in Seurat [99]. We then determined three types of DEGs: Sex-neutral DEGs (genes displaying a significant change (FDR < 0.05) between transgenic and wild type with a similar effect size in both sexes), sex-specific DEGs (genes with a significant differential expression (FDR < 0.05) in only one sex, while not approaching significance in the other sex (nominal *P*-value > 0.5)), and sex-dimorphic genes (genes with significant changes in both sexes (FDR < 0.05), but with different signs of the log fold change and a minimum log fold change difference of 0.5). In addition, we also investigated associations between gene expression and the interaction between sex and genotype to identify genes with potential sex differences affecting only the magnitude of the expression change but not the direction of the change (i.e., with the same sign of the log fold change). While we consider patterns of sex-dimorphism for the latter group of genes as less reliable than those for genes which also display a different sign of the log fold change and a minimum effect size difference, we list them as additional candidate sex-related DEGs in the Suppl. Materials and focus on the discussion of the first three groups of genes in the main manuscript. To investigate both cell type-specific and cell type-agnostic changes, we performed these analyses of DEGs both for each cell type individually and for the combination of cell types, enabling a comprehensive assessment of DEGs associated with both sex and cell type. Finally, we complemented our analyses in the THY-Tau22 model by conducting the same investigations for a human *post mortem* brain scRNA-seq dataset comparing AD patients vs. matched controls [14] and the scRNA-seq data for the Tg2576 (RRID:MGI:3710766) mouse model we have studied previously [17]. To ensure accurate assignments of standardized gene symbols for all datasets, we used the R package *HGNChelper* (version 0.8.1) for the correction of HUGO Gene Nomenclature Committee (HGNC) human gene symbols and Mouse Genome Informatics (MGI) mouse gene symbols.

To investigate the potential influence of sex on differential expression, we applied a pseudo-bulk approach with a dedicated interaction term for sex and transgene status [102]. For generating sex-related molecular signatures in each cell type, we employed the voom [103] pipeline from the R package *Limma* [104] (version 3.56.1, RRID:SCR_010943). To enhance the mean-variance trend modeling accuracy and mitigate the multiple testing correction impact, we applied the function *FilterByExpr* from the R package *edgeR* [105] (version 3.42.2, RRID:SCR_012802) with default parameters to remove lowly expressed genes. Next, we applied the trimmed mean of M values with singleton pairing (TMMwsp) method for normalization. Following this, we employed voom’s model fitting, incorporating a contrast matrix for each cell type-specific mutant-control comparison, and used an empirical Bayes estimation of standard errors [106]. DEGs were assigned as significant using stringent threshold criteria for the absolute log2 fold change (LFC > 0.25) and the adjusted p-value significance score (FDR < 0.05).

### Pathway enrichment analysis

To assess coordinated sex-dependent changes in cellular processes from public pathway databases, we conducted gene set enrichment analyses using the R package *clusterProfiler* [107] (version 4.8.1, RRID:SCR_016884) for the databases KEGG (RRID:SCR_018145) and Gene Ontology (RRID:SCR_010326). Mouse gene annotations were obtained from the R package *org.Mm.eg.db* (version 3.17.0, RRID:SCR_002811). An adjusted *p*-value below 0.05 was used as a threshold to determine significance. Finally, the enrichment analysis results were visualized using the R packages *enrichplot* (version 1.20.0), *cowplot* (version 1.1.1, RRID:SCR_018081), and *ggVennDiagram* [108] (version 1.2.3).

### Gene regulatory network (GRN) analysis

To analyze coordinated gene activity changes in regulatory sub-networks and their potential sex-dependent patterns, a gene regulatory network (GRN) was built using a dedicated network reconstruction algorithm [45] and the GeneGo MetaCore™ knowledge database (RRID:SCR_008125). This database covers curated, experimentally derived interactions between genes from the published literature. We specifically focused on curated interactions known to have a regulatory effect and clear directionality, including interactions annotated as “Regulation”, “Transcriptional Regulation”, “Influence on Expression”, “Binding”, and “co-Regulation of Transcription”. To reconstruct regulatory networks specific for each genotype, we applied a condition-specific gene regulatory network (GRN) reconstruction method [45]. This approach eliminates interactions from the initially constructed network, assembled from the knowledge base, which are inconsistent with the binary gene expression states of the transgenic and wild-type phenotypes within a genetic algorithm-based optimization procedure. Since some interactions in the MetaCore database lack information regarding their activating or inhibiting effect, the algorithm automatically infers this missing information from the available gene expression data and the known network topology.

### Analysis of network perturbations

While the GRN analysis already aids in pinpointing key regulatory genes that influence the identified DEGs, a more detailed ranking of these regulators by their relevance for controlling downstream network alterations can be obtained by computationally simulating the outcomes of altering their activity. For this purpose, we employed an algorithmic network perturbation analysis [45], which scores the potential of candidate regulator genes (called perturbagens) to reverse downstream (sex-dependent) gene expression changes. Higher scores in this analysis signify that the corresponding regulators can at least partly reverse or rescue downstream pathological expression alterations for a significant number of regulated target genes. Thus, the top-ranked genes/proteins identified by this method may serve as initial candidate drug targets for further in silico druggability analyses and subsequent experimental validation.

### Supplementary information

Reproducibility checklist

Supplementary Tables

Supplementary Figures

## Data Availability

The source code used to analyze the scRNA-seq data in the current study is available online at the GitLab repository (https://gitlab.lcsb.uni.lu/bds/thy_tau22_scrnaseq/). The entorhinal cortex dataset from THY-Tau22 mice has been deposited in NCBI’s Gene Expression Omnibus (GEO) [109] and is accessible via the GEO series accession number GSE245035.

## Code availability

The source code used to analyze the scRNA-seq data in the current study is available online at the GitLab repository (https://gitlab.lcsb.uni.lu/bds/thy_tau22_scrnaseq/). The input files used for the GRN analysis and resulting output files are available at https://webdav-r3lab.uni.lu/public/data/sbpj-x776/.
