# Supplementary material for: Single cell transcriptome analysis of the THY-Tau22 mouse model of Alzheimer’s disease reveals sex-dependent dysregulations
Source: Cell Death Discov. 2024 Mar 7;10:119. doi: 10.1038/s41420-024-01885-9 (PMC10920792; doi:10.1038/s41420-024-01885-9)
Supplement: Supplementary file 3 — Supplementary Figures [file 41420_2024_1885_MOESM3_ESM.pdf]

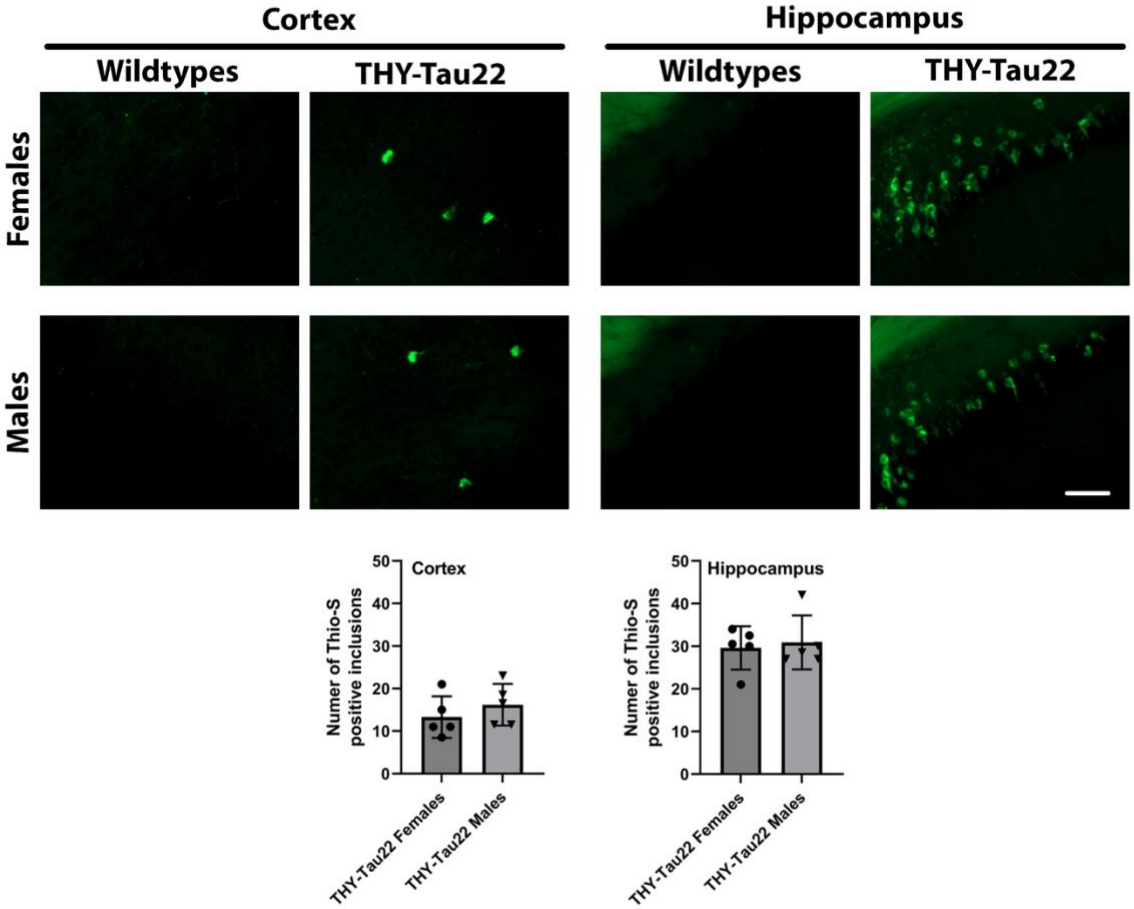

Suppl. Fig. 1: Inclusion load in the cortex and hippocampus for wildtype and THY-Tau22 mice (males and females).

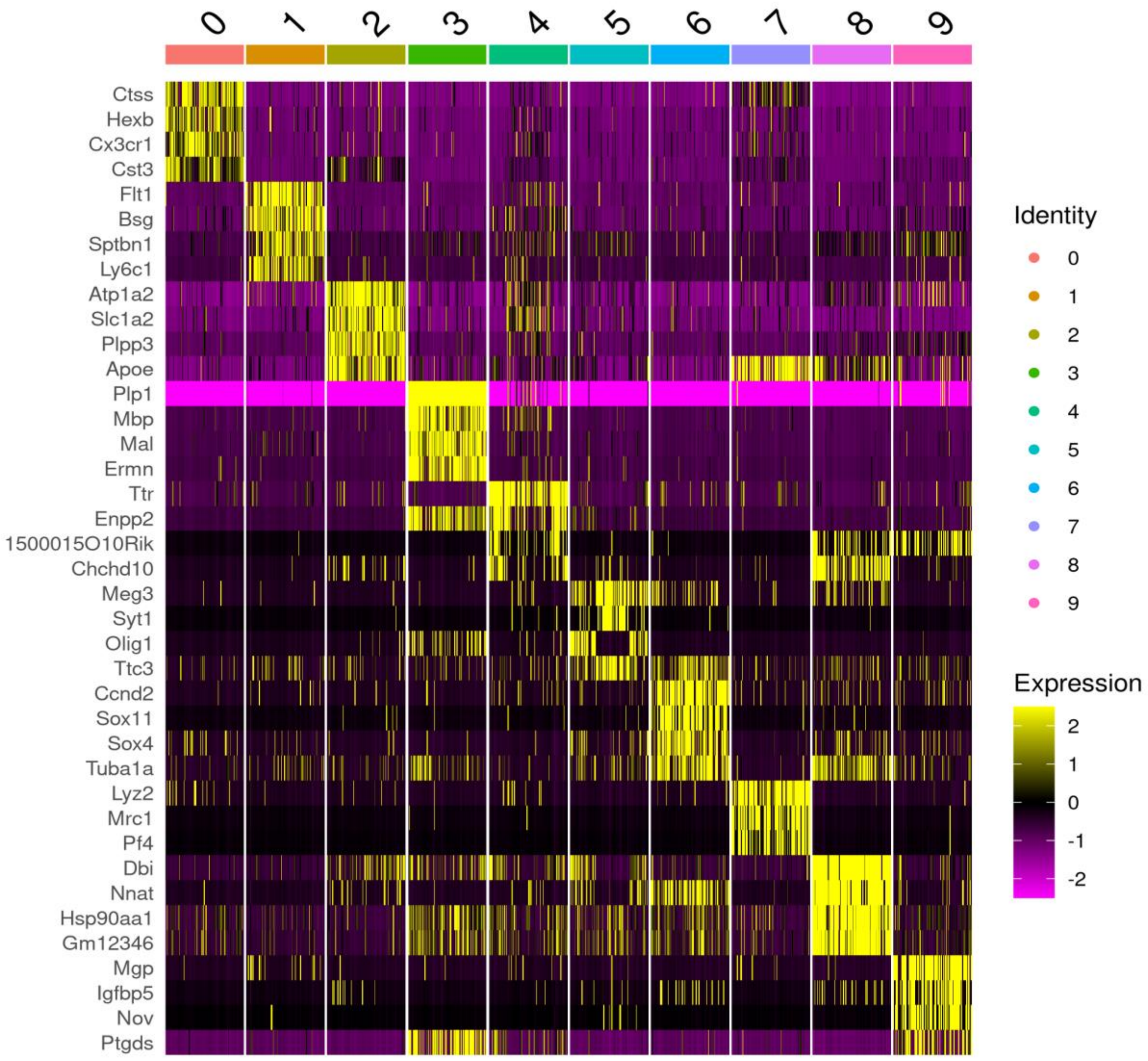

Suppl. Fig. 2: Heat map visualization of expression levels of cell type marker genes in different clusters of cells representing different cell types. Cluster numbers are shown at the top of the heat map, where clusters 0 to 9 have been annotated as microglial cells, endothelial cells, astrocytes, oligodendrocytes, OPCs, neurons, neuroblasts, macrophages, ependymal cells, and mural cells, respectively (see Suppl. Tab. 1 for more details). The marker genes shown on the left display pronounced expression levels in specific cell-type clusters (Z-score normalized expression levels are represented by a color gradient from pink to yellow, see the expression color legend on the right).
